# CHARGE syndrome-associated CHD7 acts at ISL1-regulated enhancers to modulate second heart field gene expression

**DOI:** 10.1093/cvr/cvad059

**Published:** 2023-04-13

**Authors:** Athanasia Stathopoulou, Ping Wang, Charlotte Thellier, Robert G Kelly, Deyou Zheng, Peter J Scambler

**Affiliations:** Developmental Biology of Birth Defects, UCL Great Ormond Street Institute of Child Health, 30 Guilford Street, London WC1N 1EH, UK; Department of Genetics, Albert Einstein College of Medicine, Bronx, NY, USA; School of Medical Imaging, Tianjin Medical University, Tianjin, China; Aix-Marseille University, CNRS UMR 7288, IBDM, Marseille, France; Aix-Marseille University, CNRS UMR 7288, IBDM, Marseille, France; Department of Genetics, Albert Einstein College of Medicine, Bronx, NY, USA; Departments of Neurology and Neurosciences, Albert Einstein College of Medicine, Bronx, NY, USA; Developmental Biology of Birth Defects, UCL Great Ormond Street Institute of Child Health, 30 Guilford Street, London WC1N 1EH, UK

**Keywords:** CHARGE syndrome, CHD7, Second heart field, Cardiopharyngeal mesoderm, Cardiac enhancers

## Abstract

**Aims:**

Haploinsufficiency of the chromo-domain protein CHD7 underlies most cases of CHARGE syndrome, a multisystem birth defect including congenital heart malformation. Context specific roles for CHD7 in various stem, progenitor, and differentiated cell lineages have been reported. Previously, we showed severe defects when *Chd7* is absent from cardiopharyngeal mesoderm (CPM). Here, we investigate altered gene expression in the CPM and identify specific CHD7-bound target genes with known roles in the morphogenesis of affected structures.

**Methods and results:**

We generated conditional KO of *Chd7* in CPM and analysed cardiac progenitor cells using transcriptomic and epigenomic analyses, *in vivo* expression analysis, and bioinformatic comparisons with existing datasets. We show CHD7 is required for correct expression of several genes established as major players in cardiac development, especially within the second heart field (SHF). We identified CHD7 binding sites in cardiac progenitor cells and found strong association with histone marks suggestive of dynamically regulated enhancers during the mesodermal to cardiac progenitor transition of mESC differentiation. Moreover, CHD7 shares a subset of its target sites with ISL1, a pioneer transcription factor in the cardiogenic gene regulatory network, including one enhancer modulating *Fgf10* expression in SHF progenitor cells vs. differentiating cardiomyocytes.

**Conclusion:**

We show that CHD7 interacts with ISL1, binds ISL1-regulated cardiac enhancers, and modulates gene expression across the mesodermal heart fields during cardiac morphogenesis.

## Graphical Abstract

**Graphical Abstract:**
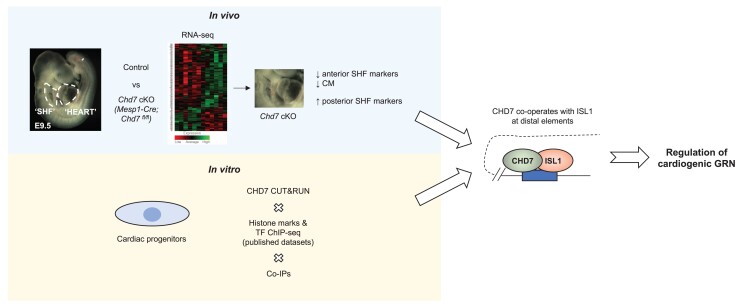

## 1. Introduction

CHARGE syndrome (Coloboma, Heart defects, Atresia of the choanae, Retarded growth and mental development, Genital anomalies, and Ear malformations) is a birth defect with significant morbidity. It is associated with deletion or point mutation of chromodomain helicase DNA binding protein 7 (CHD7), a chromatin remodeller with adenosine triphosphatase (ATPase) activity.^1^ To explore how CHD7 contributes to the morphogenesis of affected structures, animal models have been used, particularly conditional mutagenesis in mouse. Considerable emphasis has been placed on determining roles in various compartments and progenitor pools of the central nervous system. Important CHD7 functions in differentiation of neurogenic progenitors have been described,^2,3^ and CHD7 is one of the set of genes encoding chromatin modifiers where loss of function mutations are overrepresented in patients with autism (or executive dysfunction more broadly) and/or congenital heart defect.^4–6^

In terms of cardiovascular development, investigators focused on the constitutive heterozygous mouse model of the human syndrome,^7–9^ pharyngeal-epithelial-specific conditional mutants^10,11^ and neural crest cell (NCC)-specific mutants. These NCC mutants have outflow tract (OFT) and other defects.^12^ Our own work described cardiogenic mesodermal-specific (*Mesp1-Cre* conditional) null mutants (conditional knock-out, cKO) of *Chd7* which similarly had intracardiac [atrioventricular septal defects (AVSDs)] and outflow defects, together with interruption of the aortic arch, which recapitulate abnormalities seen in the human syndrome.^13^ Thus, CHD7 has multiple, important tissue specific roles that likely act through both cell autonomous and non-autonomous actions. This study aimed to identify the cardiogenic transcriptional circuitry disrupted by loss of CHD7 in cardiogenic mesoderm, and identify which, amongst these dysregulated genes, had genomic sites binding CHD7. The location of these sites provided insight into co-regulating transcriptional regulators and associated regulatory elements involved in heart development.

MESP1 is a pioneer transcription factor (TF) expressed in the cardio-pharyngeal mesoderm, a lineage forming various head and neck muscles, and much of the heart via the first and second heart fields (F/SHF).^14–16^*Mesp1-*Cre cKO mutants were embryonic lethal by E15.5 and displayed defects in both heart field derivatives.^13^ Regarding the SHF, embryos had a range of outflow defects including common arterial trunk, double outlet right ventricle (DORV) and overriding aorta. Hearts also had AVSDs, sometimes with double inlet left ventricle (the Holmes heart), and hypoplastic ventricular walls. The transcriptome of mutant embryonic day (E) 11.5 and E13.5 hearts suggested abnormal cardiomyocyte (CM) maturation and abnormal excitation contraction coupling, a defect confirmed following calcium imaging of paced primary CMs from E13.5 embryos. Together, these observations suggested a role for CHD7 in progenitors of both mesodermal heart fields providing the foundation for investigations described here.

At the biochemical level, CHD7 operates as an ATPase-dependent chromatin remodeller whereby nucleosome sliding is effected at target sites, thus secondarily affecting availability of target DNA sequences for TF binding.^17^ However, CHD7 also has non-ATPase-dependent regulatory activity^12^ possibly mediated through interactions with histone modifiers. For example, it has been proposed to be a sometime constituent of PBAF complexes in NCCs,^18^ interacting with BRG1 (SMARCA4, SWI/SNF related, matrix associated, actin dependent regulator of chromatin, subfamily a, member 4).^19,20^ Other reported interactions include the WAR complex (WD repeat domain 5 (WDR5), Absent-Small-Homeotic-2- Like protein (ASH2L), and Retinoblastoma Binding Protein 5 (RbBP5)),^21^ CHD8 (its close orthologue),^22,23^ and SMAD family member 1 (SMAD1).^24^ In this way, CHD7 has the potential to work in concert with TF binding and histone modification to modulate gene expression. It can also interact with splicing factors and abnormalities of gene splicing were reported in *Chd7* mutants.^25,26^

Both *in vivo* single cell genomics and *in vitro* differentiation of stem cells towards CMs have been helpful in elucidating gene regulatory networks (GRNs) underlying cardiac morphogenesis and disease.^27–32^ A general feature of these networks is the combinatorial binding of TFs to enhancer elements, which can result in enhancers moving through inactive/poised, and active states, and the reverse, as defined by certain histone modifications.^33^

In sum, the mutant phenotypes and functional analysis of CHD7 suggest a vital but poorly understood role for control of gene expression during cardiac morphogenesis. In this paper we explore further the role of CHD7 in cardiogenic mesoderm, identifying differentially regulated genes (DEGs) in the developing heart and caudal pharyngeal mesoderm at E9.5 upon loss of CHD7. Hybridization chain reaction (HCR) expression analysis was used to validate the targets and identify domains of expression affected by CHD7 loss. Overall, we found that certain genes expressed in the posterior SHF had increased expression, with decreased expression of genes expressed in the anterior SHF. There was a significant overlap of the DEGs with genes bound by CHD7 in murine embryonic stem cells (ESCs) differentiated to the cardiac progenitor (CP) stage. 62% of the CHD7 binding sites were >10 kb from the transcription start site (TSS) and had chromatin features associated with enhancer activity, particularly enhancer histone modification marks that change during mesoderm to CP and CP to early CM stages of mESC differentiation. Notably, there was significant overlap between ISL LIM homeobox 1 (ISL1) and CHD7 binding sites, with one such site upstream of *Isl1* itself. Moreover, CHD7 physically interacts with ISL1 in CP cells. These results provide new insights into CHD7 function in CPM, where it modulates gene expression across the mesodermal heart fields, likely through its activity at key enhancers of CPM genes.

## 2. Methods

### 2.1 Mouse lines

The mouse lines used were: *Chd7^fl^* (MGI: 4433295, *Chd7^tm2a(EUCOMM)Wtsi^*) and *Mesp1-Cre* (MGI: 2176467, *Mesp1^tm2(cre)Ysa^*). For timed matings, the date of the vaginal plug was designated as E0.5. Ear biopsies or embryonic yolk sacs were used for genotyping.

### 2.2 Ethics statement

All animal work was carried out according to UK Home Office regulations, under project license number PPL P168131D1 and conform to the guidelines from Directive 2010/63/EU of the European Parliament. The project license application was approved by the UCL Animal Welfare and Ethical Review Body before submission to the Home Office.

No anaesthetic/analgesic agents were used. Euthanasia was performed in CO_2_ chamber followed by cervical dislocation.

### 2.3 RNA sequencing

‘SHF’ or ‘HEART’ tissue was micro-dissected from E9.5 embryos and stored at −80°C, until genotyping was complete. Tissue from each embryo was processed individually and sequenced as a single sample. Each ‘SHF’ sample originated from a single embryo, and similarly each ‘HEART’ sample originated from a single heart and OFT, as described in the text. Total RNA was isolated using RNeasy Plus Micro kit, with gDNA eliminator column.

### 2.4 ‘SHF’ total RNA-seq library preparation and data analysis

Five control (*Mesp1-Cre*) and six cKO (*Mesp1-Cre; Chd7^fl/fl^*) samples were processed using the KAPA RNA HyperPrep Kit with RiboErase according to manufacturer’s instructions. ERCC RNA Spike-In Mix was added to all RNA samples. Libraries were pooled in equimolar quantities, calculated from Qubit and Bioanalyser fragment analysis, and multiplexed in the same run. Samples were sequenced on the NextSeq 500 instrument (Illumina) in a 43 bp paired end run.

Raw data were demultiplexed and converted to fastq files using Illumina’s bcl2fastq Conversion Software (v2.18) on BaseSpace. Reads were then aligned to the mouse reference genome (UCSC mm10) using STAR (v2.5.0b) on the BaseSpace RNA-seq alignment app (v1.1.0, Illumina). Reads per transcript were counted using HTSeq (RnaReadCounter by Illumina) and differential expression was estimated using the BioConductor package DESeq2 on BaseSpace app (v1.0.0).

### 2.5 ‘HEART’ mRNA-seq library preparation and data analysis

Four control (*Mesp1-Cre*) and four cKO (*Mesp1-Cre; Chd7^fl/fl^*) samples were processed using the KAPA mRNA HyperPrep kit. Libraries were pooled in equimolar quantities and sequenced on the NextSeq 500 instrument (Illumina) in a 43 bp paired end run.

We built a Galaxy pipeline which first pre-processed the data, removing any Illumina adapter contamination and poor quality base-calls using fastp (v0.20.1) before aligning reads to the mouse genome (UCSC mm10) with RNA STAR (v2.7.2b). Once aligned, reads were counted for each gene using featureCounts (v1.6.4). All sequence and annotation were obtained from the Illumina iGenomes repository. Counts were then used to perform DESeq2 differential expression analysis using the BioConductor tool (v1.3.2).

### 2.6 CUT&RUN sequencing

CUT&RUN was performed using 0.5 × 10^6^ cells (CP cells at day 5 during CM differentiation) with the CUT&RUN assay kit (CST, 86652), according to the manufacturer’s protocol. Three biological replicates were processed for each condition, using the following antibodies: anti-CHD7 (CST 6505, dilution 1/100) and anti IgG (CST 66362, dilution 1/20) as negative control. DNA fragments were purified using DNA Purification kit (CST, 14209). Library preparation was performed with the NEB DNA Ultra II assay with some deviations, as described in Supplementary material online, supplementary methods.

Library concentration was assessed with the Qubit DNA High Sensitivity assay and size distribution was assessed with the DNA 1000 High Sensitivity TapeStation assay. Samples were equimolar pooled before sequencing on the NextSeq 2000 (Illumina) according to manufacturer’s instructions. A P2 flow-cell and 100 cycle reagent kit were used to read with a 56 bp paired-end recipe along with 2 × 8 bp unique dual indexing plus an 8 bp UMI.

### 2.7 CUT&RUN data analysis

Data were UDI demultiplexed using BCL Convert (v03.75) before pre-processing with fastp (v0.20.1) to remove Illumina adapter, polyG + sequences and trim low-quality bases. Processed reads were aligned to the mouse genome (UCSC mm10) with bwa (v 0.7.17.4) before being UMI deduplicated with JE-Suite (v1.2.1). Peaks were called by MACS2^34^ (v2.1.2) for each sample with IgG as controls and overlapping peaks in at least two replicates were used as the final peaks.

### 2.8 Whole mount *in situ* HCR

Embryos E9.5 were dissected and processed for whole mount ISH. For each target mRNA a DNA probe set, DNA HCR amplifier, and hybridization, wash and amplification buffers were purchased from Molecular Instruments (https://www.molecularinstruments.com/). DNA probe sets were designed and manufactured by Molecular Instruments, using the Mus Musculus accession number for the target mRNA sequences from the NCBI database. *In situ* HCR was performed using the whole-mount mouse protocol.^35^ Following HCR embryos were counter stained with DAPI and mounted using CitiFluor AF1 mounting media into a ‘homemade chamber slide’ that provided enough space for the embryos to retain their 3D structure for imaging. Each HCR was repeated on at least four stage-matched embryos. Details on image acquisition and analysis are available in Supplementary material online, supplementary methods.

### 2.9 ESC culture and CM differentiation

Wild-type mouse ESCs were derived from C57BL6 mice in house using standard techniques.^36^ Pluripotent ESCs were maintained in serum-free conditions, in 2i medium with LIF, on gelatin-coated plates.

Mouse ESCs were differentiated to CMs as described by Wamstad *et al.*^37^ This study described four ‘key’ stages during *in vitro* CM differentiation: undifferentiated ESC (Day 0), mesoderm (MES) (Day 4; 40 h after Day 2), cardiac precursor CP (Day 5.3; 32 h after plating at day 4), and CM (Day 10). For the CUT&RUN experiments, we selected to collect the cells at Day 5, based on principal component analysis (PCA) (*Figure 2A*), as described in the results section. Our ‘Day 5’ timepoint is 24 h after plating at MES stage (Day 4) and 8 h prior to CP stage (timeline in Supplementary material online, *Figure S2A*).

Transcriptional changes during CM differentiation of our ESCs were compared with Wamstad *et al*. data and PCA showed a good matching trajectory (see Supplementary material online, *Figure S11*).

### 2.10 Co-immunoprecipitation experiments

CP cells at day 5 of *in vitro* CM differentiation were used for the co-IP experiments. 5 × 10^6^ cells were used for each antibody. Cells were lysed with IP lysis buffer (25 mM Tris-HCl pH 7.4, 150 mM NaCl, 1 mM EDTA, 1% NP-40 and 5% glycerol), supplemented with protease inhibitor cocktail (cOmplete, Roche) on ice. After sonication and centrifugation, the protein lysates were incubated overnight with 2 μg of ISL1 antibody (ab109517) or 2 μg of IgG (CST 66362) at 4°C, followed by 3 h incubation with Dynabeads protein A (ThermoFisher) at 4°C the next day.

The immunoprecipitated samples were analysed by western blot, using antibodies against CHD7 (CST 6505) and ISL1 (DSHB 39.05D).

## 3. Results

### 3.1 Global gene expression changes in E9.5 CPM of *Chd7* mutants vs. controls

**Figure 1:**
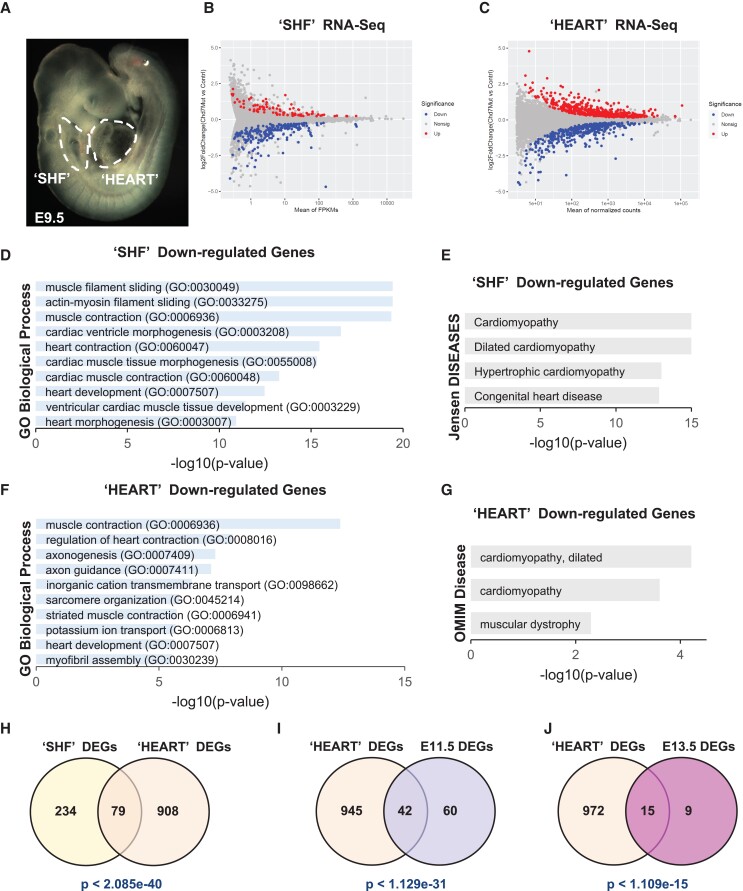
Loss of *Chd7* in CPM dysregulates the cardiogenic GRN. (*A*) Whole mount E9.5 embryo showing the regions micro-dissected for the ‘SHF’ and ‘HEART’ RNA-seq experiments. (*B–C*) MA plots showing global gene expression changes in ‘SHF’ (*B*) and ‘HEART’ (*C*) in *Chd7* cKO embryos relative to controls. Significantly changed genes with FDR < 0.1 are highlighted in red (up-regulated) or blue (down-regulated). (*D*) GO enrichment and (*E*) Jensen disease analysis for the 222 genes down-regulated in *Chd7* cKO compared to control ‘SHF’. (*F*) GO terms and (*G*) OMIM disease terms enriched in the 426 genes down-regulated in *Chd7* cKO ‘HEART’. Terms are ranked by *P*-values from the Fisher exact test, by Enrichr. (*H–J*) Venn diagrams showing the overlap between DEGs in *Chd7* cKO ‘SHF’ and ‘HEART’ samples (*H*), ‘HEART’ and E11.5 hearts (*I*), and between ‘HEART’ and E13.5 hearts (*J*). DEGs in E11.5 and E13.5 hearts were obtained from previously published data.^13^*P*-values based on hypergeometric test.

To investigate the role of *Chd7* in the CPM we performed RNA-seq on two microdissections from *Mesp1-Cre; Chd7 ^fl/fl^* (referred to as cKO) and control (*Mesp1-Cre*) embryos. The first involved the distal pharyngeal apparatus, i.e. arches two through six including the dorsal pericardial wall (DPW) which encompasses the SHF before these cells migrate to the poles of heart.^38^*In vivo,* CP cells (*Mesp1+*) found in the pharyngeal arch 2 (PA2) are reported to be a major source of cells contributing to the SHF.^39^ The region also contains less specified mesodermal multilineage primed progenitor cells (MLPs^32^). For simplicity we refer to this dissection as ‘SHF’ (we continue to use inverted commas in this denotation to recognize the presence of other cell types) as shown in *Figure 1A*. We sequenced ‘SHF’ samples from five control and six cKO single embryos. We identified 313 differentially expressed genes (DEGs, FDR < 0.1), with 91 genes upregulated and 222 downregulated in cKO ‘SHF’ (*Figure 1B*; Supplementary material online, *Figure S1A*, Supplementary material online, *Data S1*). Gene ontology (GO) analysis of down-regulated genes revealed enrichment of terms linked to the contractile apparatus, heart development, and cardiac ventricle morphogenesis (*Figure 1D*). These genes were also involved in cardiomyopathies and congenital heart disease, based on Jensen disease enrichment analysis (*Figure 1E*). By contrast, up-regulated genes were involved in glycolysis (see Supplementary material online, *Figure S1B*), expression which normally decreases as CPs differentiate.^40^

To gain insight to what cell types may be affected more functionally by *Chd7* cKO, we compared the expression of all the ‘SHF’ DEGs among cell types (i.e. clusters) identified in a previously published single cell RNA-seq data.^32^ There, the authors dissected the pharyngeal apparatus and heart from E9.5 *Mesp1-GFP* embryos *(Mesp1-Cre; ROSA26-GFP^f/+^)*, performed scRNA-seq of the *Mesp1*-expressing cells and generated clusters of the different cell types within the CPM lineage. Comparison of our ‘SHF’ DEGs with the *Mesp1-Cre* lineage at E9.5 revealed that down-regulated genes are enriched for CM cluster markers (see Supplementary material online, *Figure S1C*), suggesting that CHD7 may control normal differentiation paths during CPM development, resulting in a reduced CM population or delayed CM differentiation in the cKO embryos.

To study transcriptional changes in cells that have entered the heart (i.e. SHF-derived OFT, right ventricle (RV) and parts of the atrium, and FHF-derived left ventricle and atria), we also performed RNA-seq of the OFT and heart tissue from the same embryos, as shown in *Figure 1A* (labelled ‘HEART’). We sequenced four control and four cKO single embryos and identified 987 genes significantly dysregulated (FDR < 0.1), with 561 genes up and 426 down-regulated (*Figure 1C*; Supplementary material online, *Figure S1A*, Supplementary material online, *Data S2*). GO analysis of down-regulated genes revealed processes involved in cardiac development and muscle contraction (*Figure 1F* and *G*), terms similar to those found in ‘SHF’ DEGs. Analysis of up-regulated genes showed enrichment for extracellular matrix organization and cell migration (see Supplementary material online, *Figure S1D*). We compared DEGs of ‘SHF’ with ‘HEART’ tissue DEGs and found significant overlap (*Figure 1H*). The significance of overlap was tested using hypergeometric test and the *P*-value is provided in the figure. Most gene expression changes were in the same direction (67 of 79 common genes, 85%; Supplementary material online, *Figure S1E*).

Previous microarray analysis of E11.5 and E13.5 *Chd7 Mesp1* cKO hearts^13^ revealed abnormal CM maturation, supported by functional impairment of Ca2 + transients in paced CMs from E13.5 hearts. Our RNA-seq analysis at E9.5 showed that early CM markers were reduced in ‘SHF’ and ‘HEART’ tissue of cKO embryos, so we compared with E11.5 and E13.5 data. In E11.5 cKO hearts 102 genes were significantly changed (FDR < 0.1), with 42 (41.2%) significantly changed in E9.5 cKO ‘HEART’ (*Figure 1I*). GO (cellular components) analysis showed involvement of these genes in the sarcoplasmic reticulum membrane, endoplasmic reticulum lumen and cation channel complex (see Supplementary material online, *Figure S1F*). In E13.5 hearts 24 genes were significantly changed (FDR < 0.1), and 15 out of the 24 (62.5%) were differentially expressed in E9.5 ‘HEART’ (*Figure 1J*). This overlap was observed from E9.5 (when the embryos appear normal) until E13.5 (when the cardiovascular phenotype is apparent with external oedema and haemorrhage), suggesting that effects of gene expression may persist once CHD7 protein itself has largely disappeared from the heart at the later stage.^13^

Taken together, these data indicate that CHD7 plays a significant role in transcriptional regulation of genes important for heart tube and pharyngeal mesoderm development, with particular effects within the SHF.

### 3.2 Genome-wide analysis of CHD7 binding sites in CP cells

To identify primary and direct targets of CHD7 during the early stages of cardiac commitment and differentiation, we decided to map the genome-wide binding sites of CHD7. We used an *in vitro* approach of directed differentiation of mouse ESCs towards CMs in order to overcome technical difficulties encountered with *in vivo* derived tissues. Furthermore, *in vitro* CM differentiation allows comparison of our data with several published datasets from equivalent *in vitro* systems that map the dynamic enhancer landscapes along CM differentiation.

**Figure 2:**
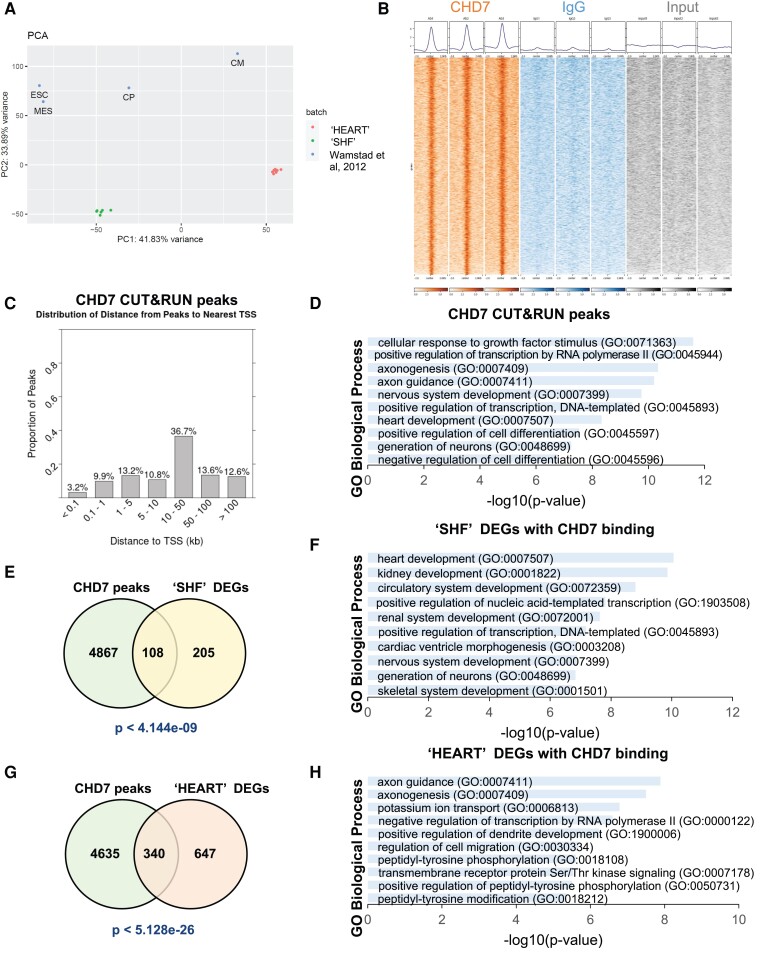
Direct targets of CHD7 in the CPM, identified by CUT&RUN. (*A*) PCA of *in vivo* control ‘SHF’ and ‘HEART’ samples compared with *in vitro* CM differentiation time points from Wamstad *et al.*^37^ based on RNA-seq data. (*B*) Read density heatmaps (+/−2 kb relative to the centre of CHD7 peaks) and density profiles of CUT&RUN-seq in 3 biological replicates. Orange, CHD7 antibody; blue, IgG antibody; grey, input. (*C*) Distribution of distances from CHD7 peaks to the nearest TSS. (*D*) GO biological processes enriched in genes associated with CHD7 peaks. Terms are ranked by *P*-value from the Fisher exact test, by Enrichr. (*E*) Venn diagram of DEGs in *Chd7* cKO ‘SHF’ with CHD7 binding, and GO terms enriched in these genes (*F*). (*G*) DEGs in *Chd7* cKO ‘HEART’ with CHD7 binding, and GO terms enriched (*H*). *P*-values of Venn diagram overlaps from hypergeometric test and *P*-values of GO analysis from Fisher exact test.

In order to identify a suitable timepoint for *in vitro* differentiation that best reflects the stage of ‘SHF’ cells *in vivo* we first compared our control RNA-seq data with the RNA-seq datasets published from Wamstad et al.^37^ In that study, the authors selected four ‘key’ stages during *in vitro* CM differentiation (ESC, MES, CP, CM) and performed RNA-seq and other genomic analysis. PCA revealed that our *in vivo* ‘SHF’ samples most closely aligned to the transition between MES and CP, but closer to CP as shown by the first principal component (*Figure 2A*). We therefore selected a time point for the *in vitro* experiments 8 h prior to their CP stage, and refer to this as ‘Day 5’ (see Supplementary material online, *Figure S2A*, protocol details in methods section). We used a CUT&RUN (‘Cleavage Under Targets & Release Using Nuclease’) technique, followed by NGS.^41,42^ This allowed us to use the low number of cells available after replating (end of day 4; 0.5 million cells for each biological replicate), and avoid limitations arising from crosslinking, sonication and solubilization.^42^ The assay was performed in three biological replicates using anti-CHD7, and IgG as negative control.

We identified 3761 peaks enriched in CHD7 Ab samples (*Figure 2B*), which were associated with 4975 genes by the software GREAT using default setting (see Supplementary material online, *Figure S2B*, Supplementary material online, *Data S3*). Using ChIP-Enrich we examined the distribution of the peaks compared to the nearest TSS (*Figure 2C*). We found 13.1% of peaks within proximal promoters (up to 1 kb from the TSS) and 26.3% within greater promoters (up to 5 kb from TSS). The majority of peaks (62.9%), however, were located over 10 kb from TSS, with 26.2% located over 50 kb from TSS (*Figure 2C*). Therefore, CHD7 was mainly associated with elements distant from the TSS but had relatively low enrichment at promoters.

GO analysis of the genes associated with the CUT&RUN peaks shows that CHD7-associated genes are enriched in several biological processes, including heart development and nervous system development (*Figure 2D*). CHD7 binding targets play significant roles in important developmental pathways such as cellular responses to various growth factors (examples in Supplementary material online, *Figure S2C*). To define direct transcriptional targets of CHD7, we intersected CHD7-bound genes with DEGs in ‘SHF’. We found 35% of cKO ‘SHF’ DEGs were bound by CHD7 (108 of 313 genes, *P* < 4.144e-09, hypergeometric test *Figure 2E*). GO analysis of these genes shows enrichment for heart development, circulatory system development, cardiac ventricle morphogenesis and skeletal system development processes (*Figure 2F*; Supplementary material online, *Figure S2D*). Both downregulated and upregulated ‘SHF’ genes independently showed significant CHD7 binding (see Supplementary material online, *Figure S2E*). The intersection of CHD7 peaks with DEGs in ‘HEART’ tissue displayed a similarly significant overlap [340 of 987 (34%) DEGs bound by CHD7, *P* < 5.12e-26, hypergeometric test, *Figure 2G*]. GO analysis revealed enrichment for axon guidance and axonogenesis, potassium ion transport, cell migration and signalling pathways involved in cardiac muscle development and function (*Figure 2H*; Supplementary material online, *Figure S2F*). The term heart development was the most significant when considering ‘HEART’ down DEGs with CHD7 peaks (see Supplementary material online, *Figure S2G*).

Taken together, this analysis indicates that a large number of our ‘SHF’ and ‘HEART’ DEGs are likely CHD7 direct targets.

### 3.3 CHD7 regulates domains of expression within the SHF

To validate expression changes and to test for alterations in the spatial expression domains as well as degree of expression, we focused upon DEGs (a) with CHD7 binding sites and (b) that have known importance within the cardiogenic GRNs (since, if CHD7 alters expression levels or domains of these genes their dysregulation likely contributes to the mesodermal loss of function phenotype). Whole mount *in situ* HCR was used to assess gene expression in *Chd7* cKO embryos at E9–9.5.

**Figure 3:**
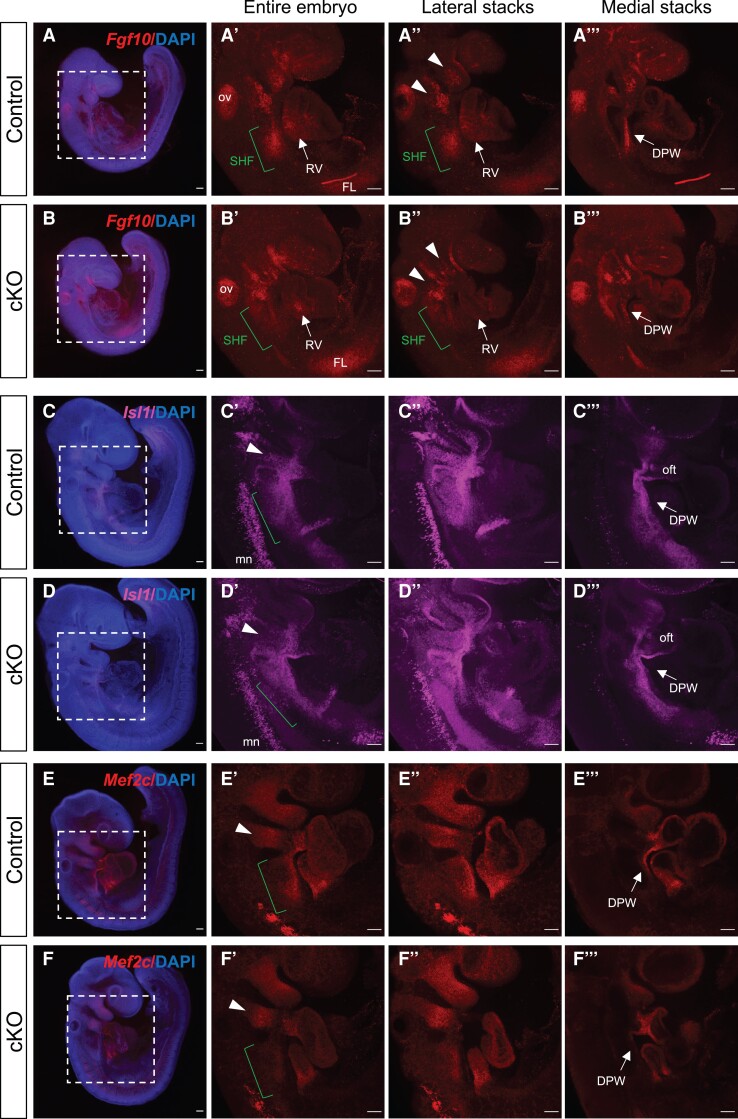
Loss of CHD7 in the *Meps1* lineage leads to reduced expression of anterior SHF markers. Whole mount *in situ* HCR staining of control and *Chd7* cKO (*Mesp1-Cre; Chd7^fl/fl^*) embryos at E9–9.5 for *Fgf10* (*A–B’’’*), *Isl1* (*C–D’’’*) and *Mef2c* (*E–F’’’*). Boxed regions in (*A*), (*B*), (*C*), (*D*), (*E*), (*F*) are shown in neighbouring panels. Confocal maximum projection of the entire embryo and selected lateral or medial z-stacks are displayed. *n* = 4. See Supplementary material online, *Figure S3* for selected stacks highlighting domain differences. Arrowheads indicate cells in branchial arches; green bracket shows the SHF region. Scale bars represent 100 μm. ov, otic vesicle; SHF, second heart field; RV, right ventricle; FL, forelimb; DPW, dorsal pericardial wall; oft, outflow tract; mn, motor neurons.

We first analysed SHF progenitor markers downregulated in cKOs. *Fgf10* is expressed in CPM including arterial pole progenitor cells and was the first molecular marker of the murine SHF.^38^ In control embryos *Fgf10* was expressed in the SHF (green bracket), the mesodermal core of the branchial arches (arrowheads) and weakly in the RV (*Figure 3A*–*A’’’*, Supplementary material online, *Figure S3A*–*A’’’*). In cKO embryos SHF expression was lost (green bracket, *Figure 3B’*–*B’’*; Supplementary material online, *Figure S3B*–*B’’*) and some minimal expression was present in the DPW in medial sections (*Figure 3B’’’*; Supplementary material online, *Figure S3B’’*–*B’’’*, quantification in Supplementary material online, *Table S1*). *Fgf10* expression in the mesoderm of the branchial arches (arrowheads) and the RV was also reduced in cKOs (*Figure 3B*–*B’’’*).

Next, we examined *Isl1*, which is expressed throughout the SHF.^43^ In control embryos *Isl1* is expressed in SHF (green bracket and DPW arrow), branchial arches (arrowheads) and OFT (*Figure 3C*–*C’’’*; Supplementary material online, *Figure S3E*–*E*’). In cKO embryos *Isl1* expression in the branchial arches was reduced, especially in the 2nd arch (arrowheads, in *Figure 3D’*–*D’’*; Supplementary material online, *Figure S3F*–*F*’, Supplementary material online, *Table S1*), but expression at the lateral part of the SHF (green bracket) and medial DPW was not severely affected (*Figure 3D*–*D’’’*).

Based on our RNA-seq data, *Hand2* and *Hand1* were reduced in cKO embryos in both dissections. In control embryos, *Hand2* is expressed in the distal portion of the branchial arches (arrowheads), in SHF (green bracket), in the OFT, and RV (see Supplementary material online, *Figure S4A*–*A’’’*), but this expression was significantly reduced in cKO embryos especially in the SHF and OFT (see Supplementary material online, *Figure S4B*–*B*’’’, Supplementary material online, *Table S1*). *Hand1* expression was also reduced in cKO embryos, mainly in the OFT (see Supplementary material online, *Figure S4C*–*D*’’, Supplementary material online, *Table S1*).

While we did not detect a CHD7 binding site at *Mef2c* this is an important part of the SHF cardiogenic GRN, and a *Mef2c* enhancer-derived CRE transgene is commonly used as mechanism for targeting this lineage.^44^ HCR revealed reduced *Mef2c* expression throughout the SHF of cKO embryos (*Figure 3E*–*F’’’*; Supplementary material online, *Figure S3C*–*D*’’’, Supplementary material online, *Table S1*).

**Figure 4:**
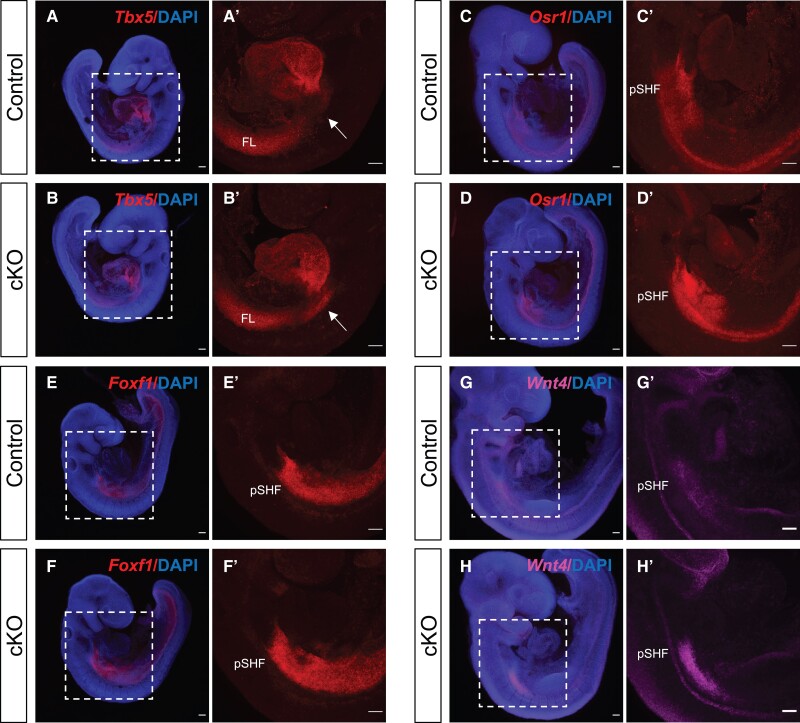
Genes expressed in the venous pole progenitor cells (pSHF) are upregulated in *Chd7* cKO embryos. Maximum intensity projections of whole mount *in situ* HCR of control and *Chd7* cKO embryos for *Tbx5* (*A–B’*), *Osr1* (*C–D’*), *Foxf1* (*E–F’*) and *Wnt4* (*G–H’*). Boxed regions in (*A*), (*B*), (*C*), (*D*), (*E*), (*F*), (*G*), (*H*) are shown in the neighbouring panel. Arrows in (*A’*) and (*B*’) indicate the expanded expression of *Tbx5* in the cKO embryos. *n* = 4. Scale bars, 100 μm. FL, forelimb; pSHF, posterior second heart field.

We then examined genes highly expressed in venous pole progenitor cells of the posterior SHF (pSHF) and upregulated in the ‘SHF’ of cKO embryos, based on the RNA-seq data. *Tbx5* is expressed in the pSHF, FHF-derived left ventricle and atria, as shown in control embryos viewed from the left side. In cKO embryos, *Tbx5* expression in the heart tube was not affected (consistent with RNA-seq data), but increased expression was observed in the posterior SHF region (*Figure 4A*–*B*’; Supplementary material online, *Figure S3G*–*H*’, Supplementary material online, *Table S1*). Whole mount HCR staining for additional genes in the pSHF—*Osr1* (*Figure 4C*–*D’*), *Foxf1* (*Figure 4E*–*F’*) and *Wnt4* (*Figure 4G*–*H’*)—showed increased gene expression levels in cKO embryos, confirming the RNA-seq data (quantification in Supplementary material online, *Table S1*).

Together these data reveal altered patterns of gene expression in the SHF such that genes expressed in arterial pole progenitor cells are downregulated and genes expressed in venous pole progenitor cells upregulated, consistent with our RNA-seq results. Other genes associated with SHF and branchial arch mesoderm were unaffected making a general cell loss unlikely. It is also important to note that these changes were subdomain specific, and not pan the *Mesp1* lineage, further supported by expression quantification of structures with no significant change (internal control tissues in Supplementary material online, *Table S1*). *Tbx1* is a SHF marker with important roles during CPM development.^14^*Tbx1* expression was not changed in cKO embryos based on RNA-seq data, and this was consistent with microarray analysis performed in *Chd7* cKO heart samples at E11.5 and E13.5.^13^ To rule out changes in domain of expression, we performed whole mount HCR to examine the spatial distribution and expression level changes of *Tbx1*; these were similar in control and cKO embryos. In particular, there was no significant difference in the expression levels of *Tbx1* + ve cells in the branchial arches and OFT or within SHF cells in the lateral and medial DPW (see Supplementary material online, *Figure S5A*–*B*’, Supplementary material online, *Table S1*). *Tbx1* is also expressed in a population of cells within the CPM recently described as MLP.^32^ At E9.5, MLP cells are localized in the lateral part of the caudal pharyngeal mesoderm, adjacent to the forming fourth arch, and these cells express *Tbx1, Aplnr* (Apelin receptor) and *Nrg1* (neuregulin),^32^ amongst other markers, none of which were altered in ‘SHF’ cKOs. Quantification of relative fluorescence intensity of *Tbx1* + ve MLP cells in control and cKO embryos showed no significant difference (see Supplementary material online, *Figure S5A*–*B*’, yellow arrow for MLPs, Supplementary material online, *Table S1*). We also stained for *Aplnr,* again finding similar expression levels in control and cKO embryos (see Supplementary material online, *Figure S5C*–*D*’, yellow arrow for MLPs, Supplementary material online, *Table S1*).

Although *Tbx1* expression appeared normal by HCR, given that a transcriptional boundary between *Tbx1*-positive progenitors at the arterial pole and *Tbx5*-positive cells at the venous pole is required for correct AV septation^45^ we examined TBX1 and TBX5 expression by immunofluorescence of sections containing the DPW. *Chd7* cKO embryos showed normal boundary formation (5/6); one embryo displayed some TBX5 positive cells in the anterior domain of the SHF. We concluded that altered TBX1:TBX5 boundary formation was not a major contributor to the AV septation defects in CHD7 cKOs (see Supplementary material online, *Figure S6*).

Together these data validate specific changes in gene expression following loss of CHD7 and highlight the role of CHD7 in patterning gene expression in the SHF.

### 3.4 CHD7 binds enhancers and co-localizes with ISL1-bound regulatory elements

#### 3.4.1 CHD7 binds cardiac enhancers

**Figure 5:**
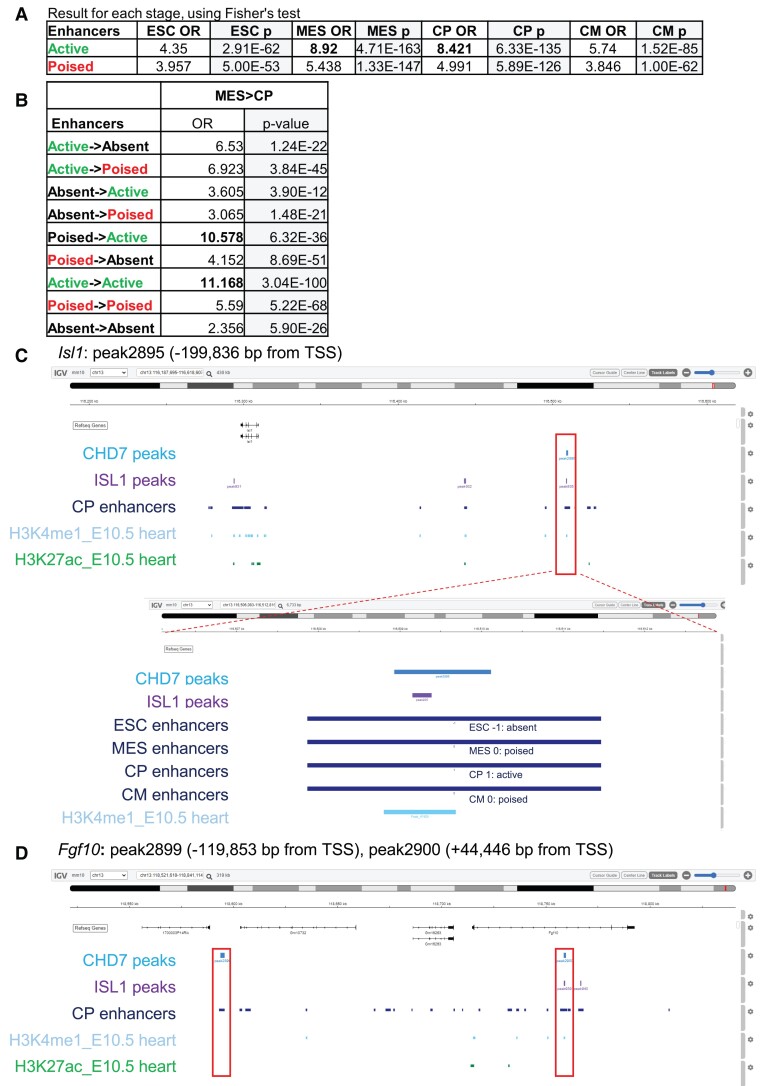
CHD7 binds cardiac enhancers in CP cells. (*A*) Overlaps between CHD7 CUT&RUN peaks and active and poised cardiac enhancers. Odds ratio (OR) and *P*-value by Fisher’s test from each stage is presented, with the overlap defined by BEDTools.^46^ (*B*) Overlap of CHD7 peaks with enhancers during MES to CP transition, based on their activity. OR and *P*-value by Fisher’s test is presented. Genome browser snapshots at *Isl1* (*C*) and *Fgf10* (*D*) loci. Snapshots include tracks from CHD7 peaks (mid blue), ISL1 peaks (purple), CP enhancers from Wamstad *et al.* (dark blue), H3K4me1 from E10.5 hearts (light blue) and H3K27ac from E10.5 hearts (green). In (*C*), CP enhancers are expanded and their activity at each stage is indicated as absent (−1), poised (0) or active (1). Details on the tracks used can be found in the methods section.

Most CHD7 binding sites (63%) are located >10 kb from TSSs, with 26% > 50 kb (*Figure 2C*). To further characterize CHD7 genomic occupancy we compared our CHD7 CUT&RUN peaks with stage-specific enhancers identified in an *in vitro* model of CM differentiation.^37^ Wamstad *et al*. classified the candidate enhancers as ‘active’ (H3K27ac+, H3K4me1+/−), ‘poised’ (H3K4me1 + only) or ‘absent’ (neither mark) at each stage (ESC, MES, CP, and CM). We found significant overlaps between our CHD7 peaks and enhancers in all categories, supported by large odds ratio (OR) and low *P*-values by Fisher's test (*Figure 5A*). The OR was particularly high for enhancer elements that are active in MES and CP, indicating the important role of CHD7 in the MES > CP transition. To further investigate, we clustered enhancers according to their states, based on marks described above, during the MES to CP transition for comparison with CHD7 peaks. We found significant overlap with all enhancer clusters, with the highest OR in the clusters that transition from ‘poised to active’ or remain ‘active’ during the MES to CP transition (*Figure 5B*).

We then examined specific CP genes dysregulated in *Chd7* cKOs (based on RNA-seq and HCR validation) that have CHD7 binding sites (see Supplementary material online, *Table S2*). *Isl1* is one example. *Isl1* expression is reduced in cKO embryos, mainly in cells migrating through the branchial arches (*Figure 3C*–*D’’’*) and has a CHD7 binding site ∼200 kb upstream of the TSS (peak2895, −199 836 bp from TSS). This site overlaps a putative CP enhancer from Wamstad^37^ also marked by H3K4me1 in E10.5 mouse embryonic heart (Bing Ren, ENCODE project)^47^ (*Figure 5C*), supporting its role as a cardiac enhancer. Detailed analysis of the Wamstad data during CM differentiation indicates that this element was not active in ESC, became poised in MES and active in CP, suggesting that activation of this regulatory element is important for the transition from MES to CP (MES > CP). This peak also had an ISL1 binding site in CP cells (see section 4.3 below). Examination of other cardiac genes with reduced expression in *Chd7* cKO (*Hand1* and *Hand2*) revealed a similar pattern, where CHD7 binding overlaps with Wamstad candidate enhancers and ENCODE histone marks in E10.5 heart samples (see Supplementary material online, *Figure S7A*–*B*), suggesting these CHD7-bound elements drive cardiac expression of these TFs.

We also interrogated genes upregulated in *Chd7* cKOs (see Supplementary material online, *Table S2*). *Tbx5* is increased in *Chd7* cKO (*Figure 4A*–*B’*) and has two CHD7 binding sites: one upstream (peak1102, −128 146 bp from TSS which overlaps with Wamstad enhancer and ENCODE histone marks), and one intronic (peak1103, +13 318 bp from TSS, no overlap with enhancer marks, Supplementary material online, *Figure S7C*).

*Foxf1* is expressed in the pSHF and its expression is upregulated in *Chd7* cKO embryos (*Figure 4E*–*F*’). *Foxf1* has a CHD7 binding site ∼100 kb upstream of the TSS (peak1795, −98 595 bp from TSS), and this site falls within a Wamstad cardiac enhancer, and is also enriched for H3K4me1, H3K27ac and K27me3 (see Supplementary material online, *Figure S8A*). *Osr1* and *Wnt4* were also upregulated in *Chd7* cKO (*Figure 4C*–*D’*, *G*–*H’*) and have CHD7 binding sites, with overlap for cardiac enhancers and ENCODE marks (*Wnt4*Supplementary material online, *Figure S8B*).

In summary, these data indicate that both up- and down- regulated genes may be targeted by CHD7, which acts primarily at cardiac enhancers dynamically regulated during cardiac morphogenesis.

Next, we decided to mine other published datasets for additional evidence of CHD7 interacting transcriptional regulators, especially in CP cells. We took three approaches: we looked for TF motifs enriched at the CHD7 binding sites (for TFs without published ChIP-seq data in CP cells), we unbiasedly searched for TFs exhibiting binding patterns significantly overlap with CHD7 (where ChIP-seq data from CP and other cardiac related cells were available), and we investigated TFs binding at the promoters of CHD7-bound genes (to account for co-regulation and looping without direct co-binding).

#### 3.4.2 Motif enrichment analysis of CHD7 binding sites

As CHD7 is not a DNA sequence specific binding factor, we used HOMER^48^ and RSAT^49,50^ to identify TF binding motifs enriched in the Chd7 CUT&RUN peaks in order to study what TFs CHD7 may cooperate with in regulating SHF development.

**Figure 6:**
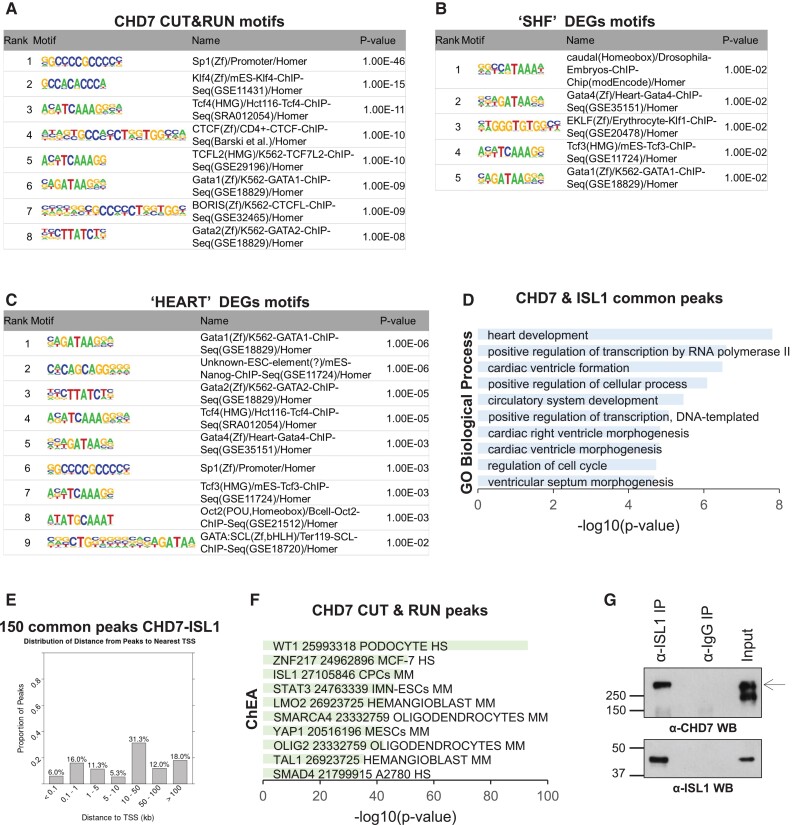
CHD7 binding sites co-localize with a subset of ISL1-bound sites and are enriched for cardiac TFs. (*A–C*) TF motifs enriched in all CHD7 peaks (*A*) and in CHD7 peaks associated with ‘SHF’ DEGs (*B*) or ‘HEART’ DEGs (*C*), as discovered by HOMER. (*D*) GO:biological processes enriched in CHD7-ISL1 common peaks, ranked by *P*-values from the Fisher exact test, by Enrichr. (*E*) Distribution of distances from CHD7-ISL1 common peaks to the nearest TSS. (*F*) Top ten transcription factors bound at the promoters of genes associated with CHD7 peaks, based on the ChEA function of Enrichr (*P*-value from Fisher exact test). HS, human; MM, mouse. (*G*) Co-immunoprecipitation with ISL1 antibody and western blot analysis for CHD7 (top) or ISL1 (bottom) in day 5 CP cells. 10% of each IP and 2.5% of input was run on 10% SDS-PAGE gel; arrow indicates CHD7 band at ∼340kD.

RSAT analysis of all CHD7 CUT&RUN peaks showed enrichment for motifs recognized by GATA and MEF2 TF families (see Supplementary material online, *Figure S9A*), further supporting a role in regulating cardiac genes, as well as SP1/KLF zinc finger motifs. HOMER analysis of the same peaks discovered 54 significantly enriched motifs (*P*-value <1e-2, *Figure 6A* top hits). CHD7 binding sites were enriched for SP11, KLF4 and GATA motifs, consistent with RSAT motifs. Moreover, HOMER showed additional enrichment of CTCF and CTCFL (BORIS) zinc finger motifs. Analysis of the CHD7 peaks associated with DEGs in ‘SHF’ (*Figure 6B*) or ‘HEART’ (*Figure 6C*) also showed significant over-representation of GATA motifs. ‘SHF’-related motifs also revealed various homeodomain TF motifs, predominantly the ‘Caudal (homeobox) motif’ as the top hit, including CDX4, CDX2, HOXD11, HOXA11 and HOXA13 TFs (based on Homer motif data).

#### 3.4.3 Sites of CHD7 binding co-localize with a subset of those of the LIM/homeodomain TF ISL1

As an unbiased approach, we used the BARTweb^51^ to search through thousands of public ChIP-seq datasets for transcription regulators with genomic binding patterns significantly similar to our CHD7 peaks. The analysis identified ISL1 (*P* = 2.2e-14, Wilcoxon rank test) among the top five regulators (the other four are chromatin factors, e.g. SMARCA4). Interestingly the similarity to published CHD7 data from non-cardiac tissues was not statistically significant (*P* = 0.17), indicating tissue specificity of CHD7 co-binding. The ISL1 ChIP-seq data were from CP cells (dataset GSE79701^52^), and we found 150 common peaks between CHD7 and ISL1 datasets. GO analysis of genes associated with these co-binding peaks showed enrichment for heart development, cardiac ventricle formation and RV morphogenesis (*Figure 6D*). We examined the distribution of CHD7-ISL1 peaks (using ChIP-Enrich) and found 33.3% within promoters (<5 kb from TSS) and 61.3% in distal elements (over 10 kb from TSS) (*Figure 6E*). Specific examples of sites occupied by both CHD7 and ISL1 include *Isl1* itself (*Figure 5C*), *Fgf10* (*Figure 5D*), *Hand1* (see Supplementary material online, *Figure S7A*), *Hand2* (see Supplementary material online, *Figure S7B*) and *Tbx5* (see Supplementary material online, *Figure S7C*).

The ISL1 connection was also supported by two additional results. The first is from RNA-seq data from ISL1 mutants.^53^ The ‘RNAseq Automatic GEO Signatures Mouse Down’ function of Enrichr showed that there was significant overlap between the ‘SHF’ down DEGs and down DEGs from (FACS sorted *Nkx2–5–GFP+*) CPCs derived by (*in vitro)* differentiation of *Isl1^−/−^* mESCs vs. control (dataset GSE80383, Supplementary material online, *Figure S9B*). Similarly, there was significant overlap of *Isl1^−/−^* CPC down DEGs and ‘HEART’ down DEGs (see Supplementary material online, *Figure S9B*). The second is from additional analysis of genes associated with CHD7 CUT&RUN peaks. Using The ChEA function within Enrichr, containing results of ChIP-seq studies, we searched for TFs bound to the promoter of genes associated with a CHD7 binding site. Third listed in terms of probability value was again the CP cell ISL1 ChIP-Seq (dataset GSE79701,^52^*Figure 6F*, enrichment *P*-value = 1.394e-49). ISL1 has been shown to bind at promoters as well as enhancers/distal elements in CP cells.^52,53^ In addition, ChEA analysis uncovered possible cooperation with WT1 (expressed in the sinus horns,^54^ 20–25% of the embryonic CMs,^55^ and important for epicardial development^56^ and EMT within mesoderm^57^).

To study whether CHD7 and ISL1 physically interact, we performed co-immunoprecipitation experiments in CP cells (day 5 during *in vitro* CM, same stage as the CHD7 CUT&RUN experiment). Immunoprecipitation with anti-ISL1 antibody followed by western blot with anti-CHD7 confirms CHD7-ISL1 protein interaction (*Figure 6G*).

Focusing on the subset of genes with CHD7 binding sites and differentially expressed in ‘SHF’ (see Supplementary material online, *Figure S9C*), and similarly for the ‘HEART’ dissection (see Supplementary material online, *Figure S9D*), we found that promoters at these genes were significantly enriched for proteins of the polycomb group complex (PcG). Unsurprisingly, ISL1 was also enriched at these promoters (‘SHF’: *P*-value = 1.24e-8, adj. *P*-value = 3.26e-7, Supplementary material online, *Figure S9C* and ‘HEART’: hit *P*-value = 1.669e-16, adj. *P*-value = 1.519e-14, Supplementary material online, *Figure S9D*).

Finally, one well studied enhancer provides an informative example. *Fgf10*, which is downregulated in *Chd7* cKO embryos (*Figure 3A*–*B*’*’’*), is associated with two CHD7 CUT&RUN peaks: one upstream (peak2899, −119 853 bp from TSS) and one intronic (peak2900, +44 446 bp from TSS). Both the upstream and intronic peaks coincide with cardiac enhancer elements^37^ and E10.5 heart H3K4me1 marks (*Figure 5D*). Interestingly, the intronic peak ‘peak2900’ is located within a characterized *Fgf10* enhancer, a 1.7 kb regulatory element important for the regulation of *Fgf10* expression in SHF vs. differentiated CMs and left ventricle.^58^ Notably, the intronic peak also contains TBX1 and NKX2.5 binding sites as well as for ISL1. Moreover, the upstream peak ‘peak2899’ is conserved in mammals and maps to the location of the *Fgf10* ‘enhancer trap’ cassette *Mlc1v-nLacZ-24* that originally led to the identification of the SHF as a source of CPs for the RV and the OFT myocardium.^38^

Thus, collectively our analysis implicates CHD7 in the transcriptional control of ISL1-regulated cardiogenic TFs, and the complex switch of *Fgf10* expression during heart development.

## 4. Discussion

Given the varied and severe heart malformations seen in the *Mesp1-Cre; Chd7* cKO embryos we set out to identify genes whose dysregulation are likely to contribute to these phenotypes. We paid particular attention to genes whose loci were bound by CHD7 as these would more likely be direct targets. Examination of existing datasets, TF binding motif analysis and gene ontology were employed to assess the most important TF interactions and pathways involved.

Although our E9.5 ‘SHF’ bulk RNA-seq contained cells outside the *Mesp1* lineage e.g. pharyngeal epithelia, the dissection to remove the first pharyngeal arch and neural tube overlying the caudal arches should enrich for CPM while not introducing noise from exposure to FACS purification. This dissection includes the DPW from which SHF cells migrate to the arterial poles, and a progenitor cell pool recently described and characterized by expression of *Aplnr* and *Tbx1.*^32^ The ‘HEART’ dissection would contain cells from both heart fields, but at a more advanced stage of differentiation, including early CMs, thus likely capturing longer term and late effects. A significant number of genes were concordantly differentially expressed across both data sets. Included in these were *Sema3a* and *Sema3c* (HCR staining with quantification in Supplementary material online, *Figure S10*), both genes reported to have decreased expression in later stage *Mesp1* cKOs,^13^*Chd7* heterozygous whole embryos,^59^ and, for *Sema3a* only, a Xenopus model of CHARGE syndrome.^60^ Notably, the Semaphorin3 receptor *PlexinA2* is a BRG1:CHD7 target in NCCs.^19^ CHD7 has similar phenotypes in the NCC and mesoderm conditional mutants, in particular both have 4th PAA and OFT septation defects; mesodermal mutants have more severe AVSDs. It is known that the SHF and NCC ingressing into the OFT reciprocally interact.^61^ One possible mechanism is via Semaphorin signalling. For example, NCC derived-Sema3c is required for EMT during OFT septation,^13^ and the Sema3 receptor PLXNA2 is similarly required in the cardiac NCC.^62^

We compared the E9.5 data with E11.5 and E13.5 transcriptomic data from *Mesp1* cKOs that we published previously. Of interest, the failure of CM differentiation culminating in abnormal coupling of calcium signalling to pacing^13^ was foreshadowed by the decreased expression of early CM markers shown most evidently in Supplementary material online, *Figure S1C*. Some genes were concordantly differentially expressed in both E9.5 dissections and whole hearts at E11.5 (*Figure 1I*). The reduction of markers associated with CM function was particularly striking.

To confirm RNA-seq results and to examine overall vs. region-specific changes in gene expression we selected candidate DEGs for validation on the basis of CHD7 binding sites at the genomic locus (discussed below) or because they mark specific cell populations in the case of *Mef2c*, and the control (expression unchanged) genes *Tbx1* and *Aplnr*. The expression levels of SHF arterial pole progenitor cell markers *Fgf10*, *Mef2c*, and *Hand2* were reduced in cells in the medial and lateral DPW as well as the PAs, while *Isl1* expression was only reduced in the cells migrating through the PAs and not cells in the DPW. Of the pSHF genes *Wnt4* was increased across its SHF domain, whereas the picture for *Tbx5* was more complicated. Medial expression in the DPW, where it establishes a boundary with *Tbx1* in concert with retinoic acid signalling, did not appear affected. However, the *Tbx5* expression domain was expanded caudally on the lateral side of DPW. Unaltered expression of the SHF MLP pool marker *Aplnr*^32^ and *Tbx1,* which is expressed across the anterior and posterior SHF,^58,63^ indicate diminished expression of the aSHF genes listed above rather than loss of cells in which the genes are expressed.

In *Mesp1Cre* cKO mutants we see malformations of the OFT and interrupted aortic arch, which will likely be due to cell autonomous alterations of gene expression in the SHF (e.g. several of the differentially regulated TFs, especially *Isl1*, *Mef2c* and *Hand2*) and/or noncell autonomous effects on cardiac NCCs (e.g. via semaphorins, FGF10). Disturbance of relative levels of expression of anterior vs. posterior SHF genes could affect both cardiac outflow and inflow patterning. The AVSDs seen in *Mesp1* cKOs may be secondary to anomalous SHF or FHF contributions, or both. SHF derivatives are found in the dorsal mesenchymal projection (DMP), a structure vital for atrial and atrioventricular septation. A boundary between TBX1-positive arterial pole and TBX5-positive venous pole progenitors is vital for the DMP contribution^45^ but is established normally in *Chd7* cKOs. Several genes specifically involved in AV canal morphogenesis showed altered expression e.g. *Wnt4* (HCR confirmed, with CHD7 binding sites). *Wnt2*, which also displayed increased expression in the ‘SHF’ sample, is expressed specifically in the developing inflow tract mesoderm and required for its remodelling, although we could not obtain good quality HCR data for this gene. GO analysis revealed WNT pathway as an enriched term for genes associated with CHD7 CUT&RUN peaks (see Supplementary material online, *Figure S2C*), and also for the intersect of genes with CHD7 peaks and differentially expressed in ‘SHF’ (see Supplementary material online, *Figure S2D*). *Sema6D*, which had reduced expression in both ‘SHF’ and ‘HEART’ dissections, is required for atrioventricular septation, compact-layer expansion and trabeculation.^64,65^*Tbx2*, with reduced expression in mutant hearts, is similarly required for AVS formation.^66^ TBX5 target genes *Osr1,*^67^*Fendrr* and *Foxf1* are each upregulated. The TSS for the non-coding RNA *Fendrr* is approximately 1.3kbp upstream of *Foxf1.*^68^ Thus either or both genes might be coregulated by CHD7 binding to the site in Supplementary material online, *Figure S6A*. *Osr1* and *Foxf1* are both involved in remodelling the AV canal via a hedgehog cascade,^68^ and *Fendrr* is itself vital for normal heart development.^69^ Myosin heavy chain gene (*Myh*) mutations have also been described in human and murine heart defects (reviewed in^70^). Thus, a substantial number of genes orchestrating atrioventricular septation are dysregulated in *Mesp1* cKOs.

As there is substantial extant data on regulatory elements modulating cardiac mesoderm differentiation, our results were compared to genome wide datasets to establish whether CHD7 mainly acted at known regulatory sites and TFs likely to be involved in co-regulation. There was significant overlap of DEGs and the presence of one or more CHD7 binding sites at that locus. The majority of binding sites were at sites where histone modifications suggested an enhancer element, either in differentiating mESCs,^37^ or in murine E10.5 whole hearts.^71^ In the differentiating mESCs, the most significant overlap of CHD7 was at sites with dynamically changing histone modifications during the mesodermal to CP or CP to CM transitions.

Another significant finding was the overlap with ISL1 binding as determined by ChIP-seq in CP cells.^52^ One of these binding sites was at *Isl1* itself, was conserved in human sequence and showed dynamic histone mark transitions during CP differentiation from mESCs (*Figure 5C*). Others included *Fgf10, Hand1, Hand2, Tbx5,* and *Sema6d*. The effect of loss of *Isl1* in developing cardiac lineages is also similar to loss of *Chd7* in both our ‘SHF’ and ‘HEART’ dissections from the point of DEGs. Interestingly, 25% of CHD7 binding sites were >50 kb from the TSS; it is notable that a study in β-cells of the pancreas, ISL1-enhancer-promoter loops had a median enhancer-promoter distance of 165kb.^72^ ISL1 is a pioneer factor capable of binding compacted chromatin. A recent study described how ISL1 cooperates with a BRG1-BAF60c-based SWI/SNF complex to effect epigenetic regulation of the chromatin landscape of genes in the cardiogenic GRN, including CM structural genes expressed once *Isl1* itself is extinguished.^53^ In this paper, four of eleven cardiac GRN genes shown to be downregulated in *Isl1-Cre^+/−^/Brg1^fl/+^* and *Isl1-Cre^+/−^/Brg1^fl/fl^* dissected E10.5 OFT and RV (SHF derivatives) (*Figure 5I* in ref.^53^) were similarly dysregulated in our E9.5 ‘SHF’ cKO dissections (*Hand2*, *Mef2c*, *Tbx20* and *Ttn*). Thus, these widespread effects mirror what we observe with CHD7 and support a CHD7-ISL1 co-operation, which is further supported by co-immunoprecipitation of the two proteins in CP cells (*Figure 6G*).

There are indications an ISL1-CHD7 relationship exists outwith the cardiovascular system. Hurd *et al.* suggest CHD7 regulates *Fgf10* expression in neuroblasts during inner ear neurogenesis through one or both of inhibition of *Tbx1* and activation of *Isl1/NeuroD/Ngn1.*^73^ In zebrafish, knockdown of CHD7 produces abnormal organization of cranial motor neurons tagged with GFP driven by ISL1 regulatory elements.^74^ Moreover, we performed HOMER motif enrichment analysis of previously published CHD7 ChIP-seq data from cerebellar granule cells^75^ and found significant enrichment for the neuronal ISL1 motif (with *P*-value 0.0001). We examined CHD7 binding sites for specific DNA sequence motifs. The GATA motif was the most over-represented. Notably, it has been shown that CHD7 interacts directly with GATA4 protein in CP cells derived from human iPS cells,^76^ that ISL1 and GATA4 often co-occupy sites with open chromatin,^53^ and ISL1 and GATA4 co-operate in regulation of *Fgf10.*^77^ Also of interest was the over-representation of CTCF and CTCFL (BORIS) binding sites. CTCF/L are 11-Zn finger proteins that bind to insulator elements, interfering with enhancer-promoter interactions thereby repressing or activating transcription at long range, and establishing domains permissive (or not) for transcription. Notably, CHD8 directly interacts with CTCF, and ChIP of CHD8 (the most closely related chromo-domain remodeller) showed it localized to many of the same sites as CTCF in Hep3B cells.^78^ CDX motifs were prominently over-represented; CDX TFs have important roles during early mesoderm development; more specifically CDX2 interacts and colocalizes with BRG1 (catalytic subunit of SWI/SNF) at several cardiogenic loci.^79^

As enhancers are known to loop to promoter sequences in a higher order chromatin structure, we wondered what transcriptional regulators might be over-represented at DEGs bound by CHD7: as CHD7 is likely to be part of multimeric complexes first bound at enhancers its fixation to promoters during our experiments might be weakened leading to underrepresentation. Here too, we found the presence of ISL1, but the most intriguing observation was the presence of many members of the polycomb group of proteins (PcG). Polycomb proteins are generally, but not always, associated with repression of transcription and H3K27me3 modification especially at genes regulating development. While we have not taken the observation further it is worthy of additional study since the fly ortholog of CHD7 (and CHD8), kismet, is known to antagonize PcG-mediated repression during development.^80,81^ In G4 human medulloblastoma cells, CHD7 creates a less accessible chromatin conformation thereby preventing BMI1 repression of DUSP4,^82^ and more recent studies demonstrated the presence of a CHD7-BMI1-MAPK regulatory axis,^83^ so one specific example of PcG-CHD7 interaction already exists.

**Figure 7:**
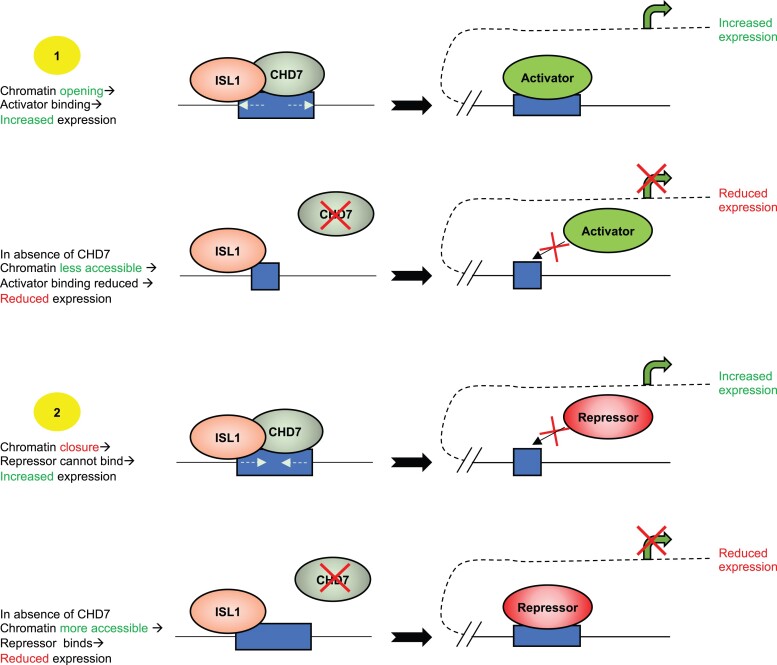
CHD7—proposed modes of action using *Fgf10* enhancer as example. (An extended version of the model presented by Watanabe *et al*.^58^) As mentioned above, while CHD7 may participate in recruitment of histone modifiers and splicing the model focuses on its ATPase dependent nucleosome remodelling activity.^17^ Depending upon additional specific transcriptional regulators, opening of chromatin could be associated with either up or down regulation, and conversely closure could again be associated with increased or decreased target expression. In the context of the regulation of *Fgf10* expression specifically via the intragenic enhancer bound by ISL1 (and TBX1), and its repression via NKX2.5, absence of CHD7 would be predicted to interfere with this balanced control as cells progress from a progenitor to differentiated state.

As an insight into how CHD7 may function at a specific regulatory element the intragenic CHD7 binding site within *Fgf10* is of especial interest, *Fgf10* expression being downregulated in the aSHF of CHD7 cKOs. This element is instructive since it contains binding sites for ISL1, NKX2.5, GATA and TBX1. Enhancer transgenesis showed it activates *Fgf10* expression in the SHF via ISL1 activity, but represses, via NKX2.5, in myocardium.^58^ CHD7 also binds an upstream FGF10 element (*Figure 5D*) and this site is coincident with a nLacZ enhancer trap that was used in the initial description of a ‘second heart field’.^38^ This binding site shows mammalian sequence conservation and has GATA and MEF2a/c motifs. As an ATP-dependent nucleosome sliding protein CHD7 binding could modulate transcription in various ways as depicted in the context of ISL1 co-regulation in the model of *Figure 7*.

## 5. Conclusion

Our data reveal that CHD7 fine-tunes the expression of genes in the cardiogenic GRN during cardiac morphogenesis. This includes critical TFs such as *Isl1, Hand1, Hand2, Tbx5, Foxf1, Osr1* as well as signalling proteins such as *Fgf10*, *Wnt4*, and Semaphorins. We present evidence that CHD7 acts at sites bound by the pioneer TF ISL1, and at enhancers >50 kb from the TSS, a feature also noted for ISL1. CHD7 physically interacts with ISL1 in CP cells, we therefore suggest important co-operation during mesodermal through CM differentiation. TF-CHD7 interactions may be important where dynamic changes in expression level are required during mesodermal differentiation as exemplified by *Fgf10* expression within a SHF domain and its repression in FHF lineages contributing to the left ventricle. In conclusion, it is clear that CHD7 has vital functions in the SHF as well as mesoderm more generally during heart morphogenesis. Issues such as CTCF, CHD8, and polycomb interactions may prove fruitful avenues for future research.

## Supplementary material

Supplementary material is available at *Cardiovascular Research* online.

## Supplementary Material

cvad059_Supplementary_Data

## Data Availability

The data that supports the findings of this study are available from the corresponding author upon reasonable request. Sequencing data have been deposited in NCBI Gene Expression Omnibus and are accessible through GEO Series accession number GSE229630.

## Notes

Translational perspective CHD7 is a chromatin remodeller haploinsufficient in CHARGE syndrome and implicated in autism spectrum disorder and various cancers. Heart defects in the syndrome are recapitulated by murine loss-of-function in two lineages, neural crest, and cardiopharyngeal mesoderm (CPM). CHD7 regulates vital cardiogenic genes via binding predominantly to enhancers distant from the target gene at sites often shared with the pioneer transcription factor ISL1. CHD7 bound enhancer elements show highly dynamic switching of histone modifications during the mesodermal to cardiac progenitor cell transition. Thus, manipulation of CHD7 activity may assist in directed differentiation of distinct cardiovascular progenitors for use in regenerative/repair therapeutics.
