# Corrigendum to: CHARGE syndrome-associated CHD7 acts at ISL1-regulated enhancers to modulate second heart field gene expression

**DOI:** 10.1093/cvr/cvad094

**Published:** 2023-07-03

This is a corrigendum to: Athanasia Stathopoulou and others, CHARGE syndrome-associated CHD7 acts at ISL1-regulated enhancers to modulate second heart field gene expression, *Cardiovascular Research*, Volume 119, Issue 11, August 2023, Pages 2089–2105, https://doi.org/10.1093/cvr/cvad059

Upon publication, affiliation 4 in this manuscript was incorrectly given as ‘Developmental Biology, Institute of Marseille, CNRS UMR 7288, IBDM, Marseille, France’.

The affiliation has now been corrected to read as follows: ‘Aix-Marseille University, CNRS UMR 7288, IBDM, Marseille, France’.

