# Supplementary material for: CHARGE syndrome-associated CHD7 acts at ISL1-regulated enhancers to modulate second heart field gene expression
Source: Cardiovasc Res. 2023 Apr 13;119(11):2089–105. doi: 10.1093/cvr/cvad059 (PMC10478754; doi:10.1093/cvr/cvad059)
Supplement: cvad059_Supplementary_Data [file cvad059_supplementary_data.zip › Supplementary Figures_updated.pdf]

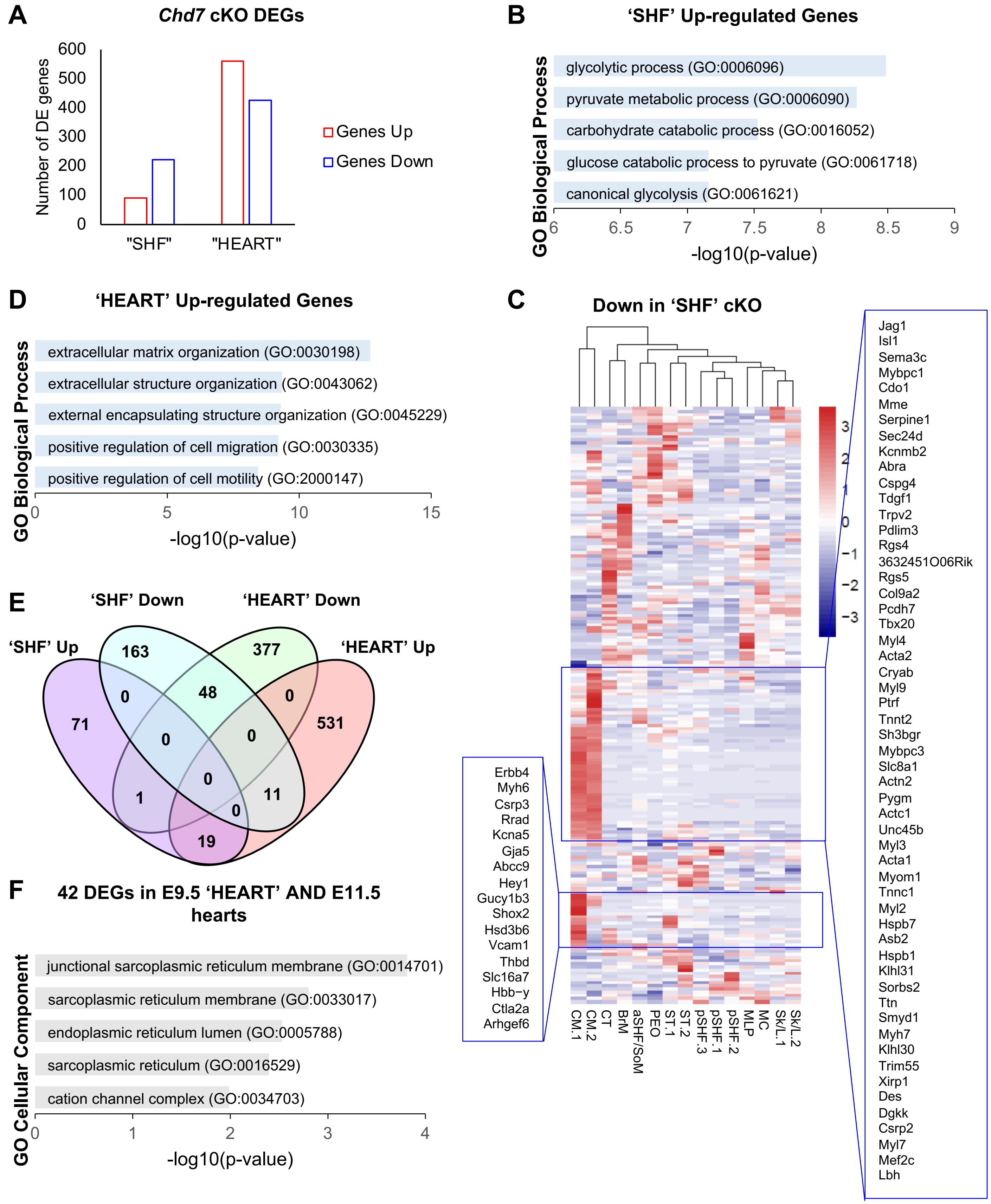

Supplemental figure S1, related to figure 1: Reduced CM markers in unbiased analysis of *Chd7* cKO 'SHF' downregulated genes.

(A) Bar graph showing the numbers of DEGs in 'SHF' and 'HEART' tissue of *Chd7* cKO embryos. (B, D) GO terms enriched in genes up-regulated in *Chd7* cKO 'SHF' (B) and 'HEART' (D) ranked by p-value from the Fisher exact test, by Enrichr. (C) Comparison of down-regulated genes in *Chd7* cKO 'SHF' with previously published single cell RNA-seq data from Mesp1-expressing cells[32]. Clusters of the different cell types are shown at the bottom of the heatmap and they are the same as in the original publication. Selected genes enriched in CM clusters are presented on the right and left side of the heatmap. Row indicates the mean gene expression in each cluster (blue to white, to red; low to high). (E) Venn diagram showing the overlap of genes up- or down-regulated in cKO 'SHF' and 'HEART'. (F) GO:cellular component analysis of common DEGs in cKO 'HEART' and E11.5 hearts, related to figure 1I.

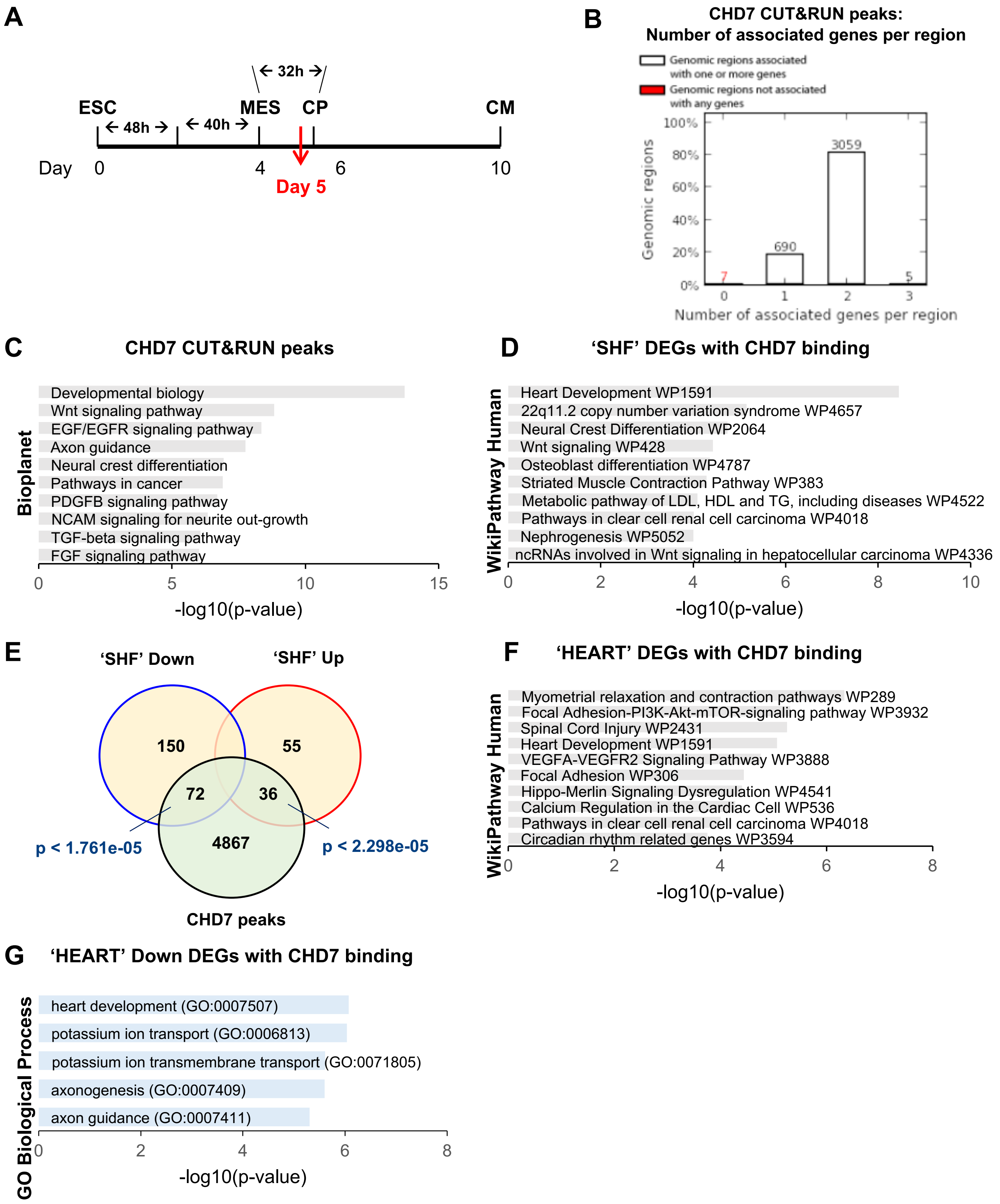

Supplemental figure S2, related to figure 2: CHD7 direct targets.
(A) Timeline of cardiomyocyte differentiation protocol, showing 'key' stages described by Wamstad et al [37]. ESC, embryonic stem cell; MES, mesoderm; CP, cardiac precursor and CM, cardiomyocyte. Based on PCA (figure 2A) we selected 'Day 5' as the appropriate stage for the in vivo 'SHF' samples. 'Day 5' is exactly 24 hours after the MES stage at day 4, when the cells are plated onto gelatin-coated plates. (B) Bar graph showing the number of genes associated with CHD7 peaks. (C) Bioplanet pathways enriched in genes associated with CHD7 peaks. Pathways enriched in 'SHF' (D) or 'HEART' (F) DEGs with CHD7 binding based on WikiPathway human enrichment. Terms are ranked by p-values from the Fisher exact test, by Enrichr. (E) Venn diagram displaying overlap of CHD7 peaks with downregulated (down) and upregulated (up) genes in 'SHF'. p-values are from hypergeometric test. (G) GO biological processed enriched in genes downregulated in 'HEART' with CHD7 binding, ranked by p-values (from Fisher exact test).

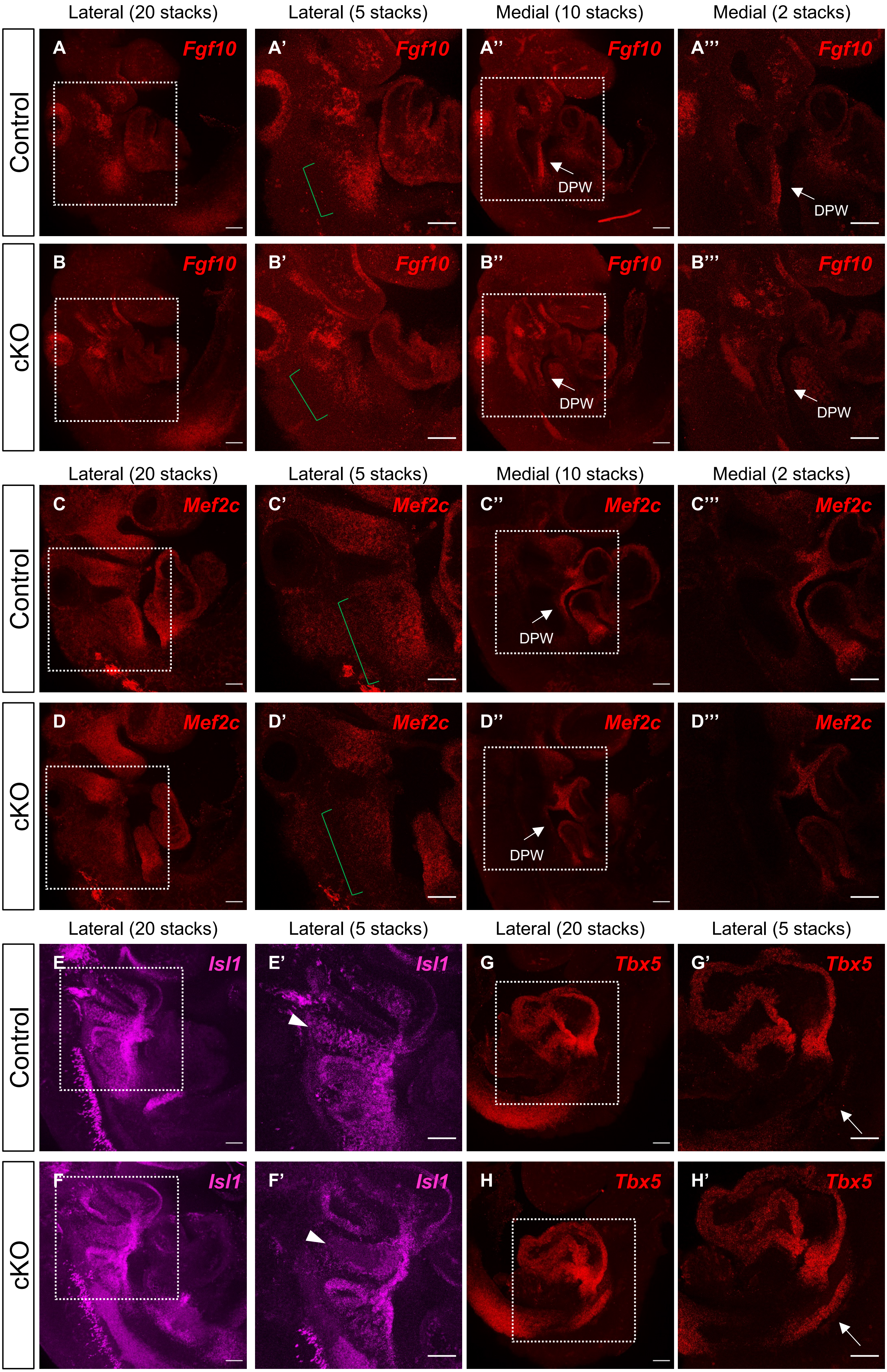

Lateral (20 stacks)
Lateral (5 stacks)
Medial (10 stacks)
Medial (2 stacks)
Control
cKO
A
Fgf10
A'
A''
A'''
B
B'
B''
B'''
DPW
Lateral (20 stacks)
Lateral (5 stacks)
Medial (10 stacks)
Medial (2 stacks)
C
Mef2c
C'
C''
C'''
D
D'
D''
D'''
Lateral (20 stacks)
Lateral (5 stacks)
Lateral (20 stacks)
Lateral (5 stacks)
E
Isl1
E'
G
Tbx5
G'
F
F'
H
H'

Supplemental Figure S3, related to figures 3 and 4: Additional views and 'virtual' sections of the embryos presented in figures 3 and 4.
Whole mount *in situ* HCR staining of control and *Chd7* cKO (*Mesp1-Cre; Chd7*$^{fl/fl}$) embryos at E9-9.5 for *Fgf10* (A-B'''), *Mef2c* (C-D'''), *Isl1* (E-F') and Tbx5 (G-H'). Some of the thick 'virtual' sections are also presented in the main figures. Boxed regions in are shown in neighbouring panels.
Confocal maximum projection of selected lateral or medial z-stacks are displayed, with the number of stacks used indicated on top of each image. The 'step' between the different z-stacks is 3.25μm. n=4.
Arrowheads indicate cells in branchial arches, green bracket shows the SHF region. SHF, second heart field; DPW, dorsal pericardial wall. Scale bars represent 100μm.

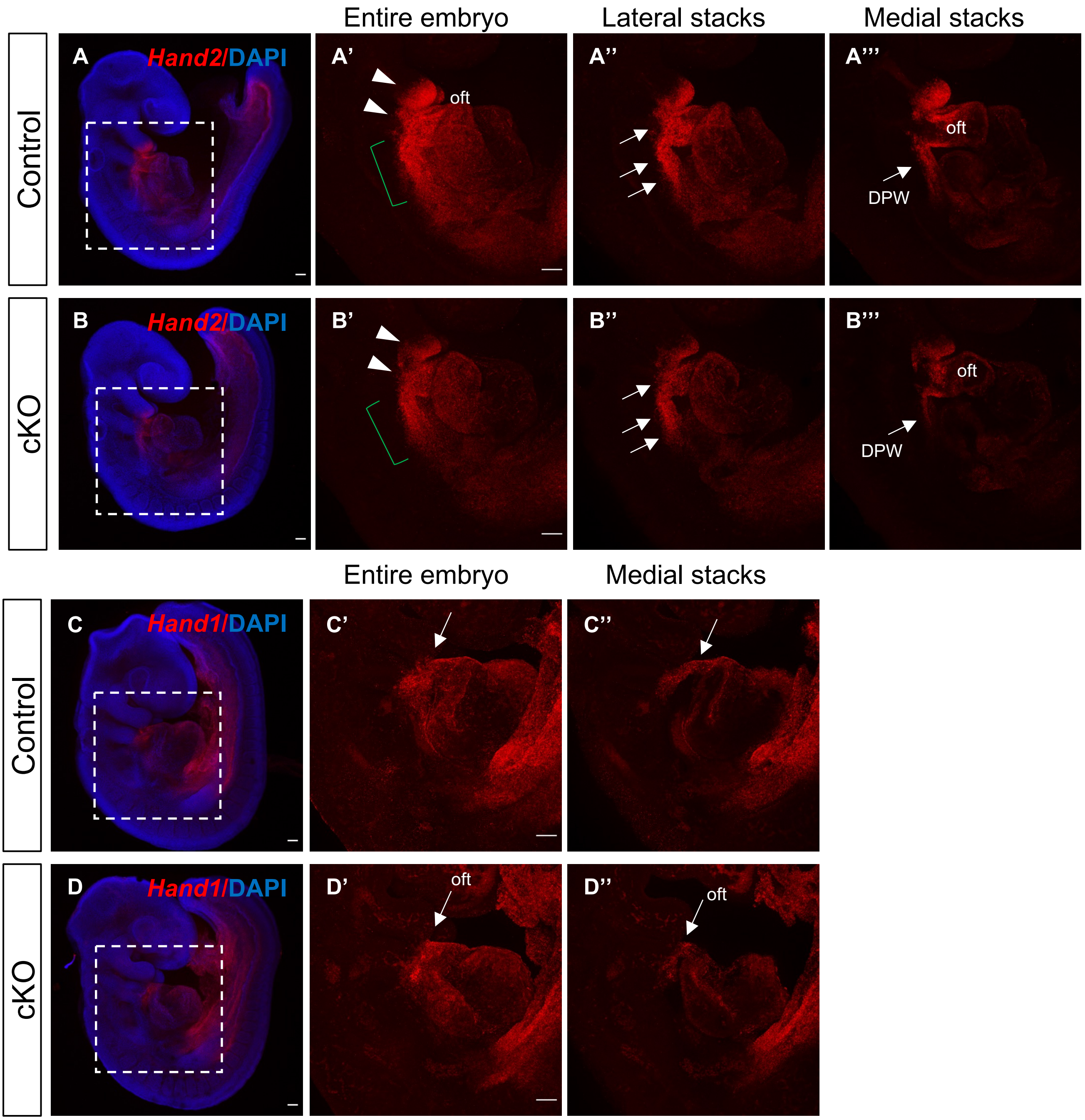

Supplemental Figure S4: Expression of *Hand1* and *Hand2* is reduced in *Chd7* cKO embryos.
Whole mount in situ HCR staining of control and *Chd7* cKO embryos at E9.5 for *Hand2* (A-B''') and *Hand1* (C-D'''). The entire embryo and selected lateral and medial stacks are presented as maximum z projection. n = 4. Scale bars, 100µm. DPW, dorsal pericardial wall; oft, outflow tract.

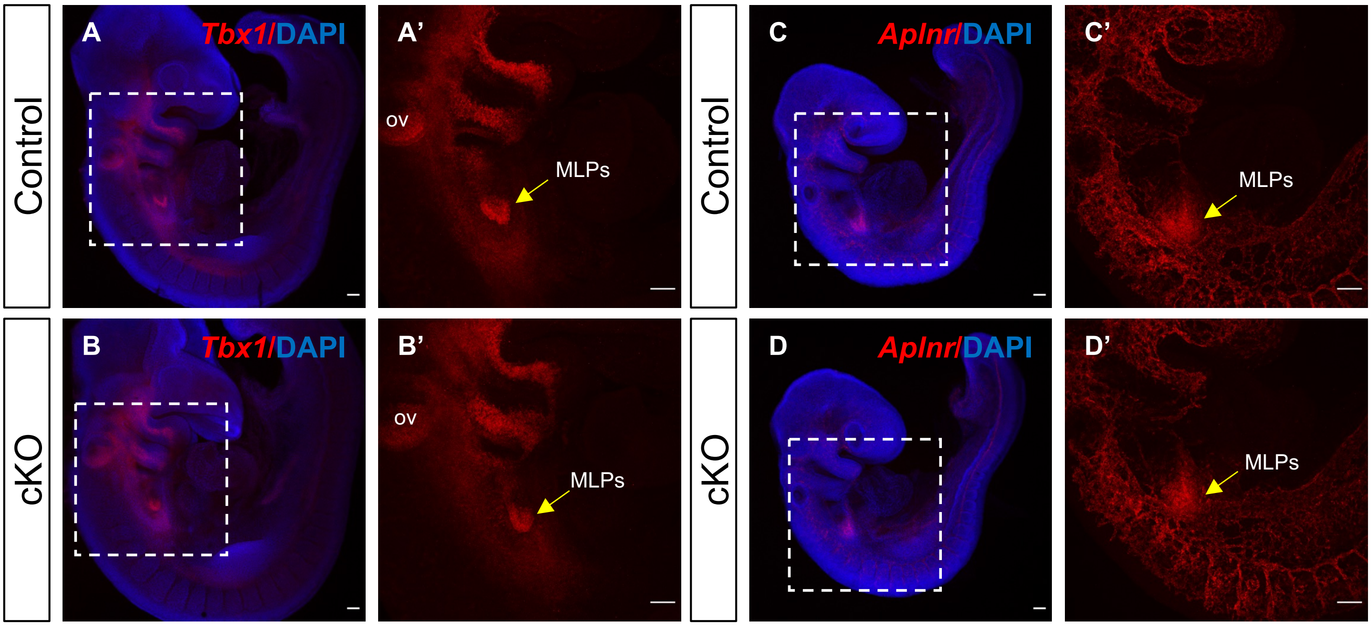

Supplemental Figure S5: Distribution and expression of *Tbx1* and *Aplnr* is not altered in *Chd7* cKO embryos.
Whole mount *in situ* HCR staining for *Tbx1* (A-B') and *Aplnr* (C-D') in control and *Chd7* cKO embryos. Maximum intensity projections of the entire embryos are presented. Yellow arrows highlight the MLP cells. n=4.
Scale bars,100µm. ov, otic vesicle; MLPs, multilineage primed progenitors.

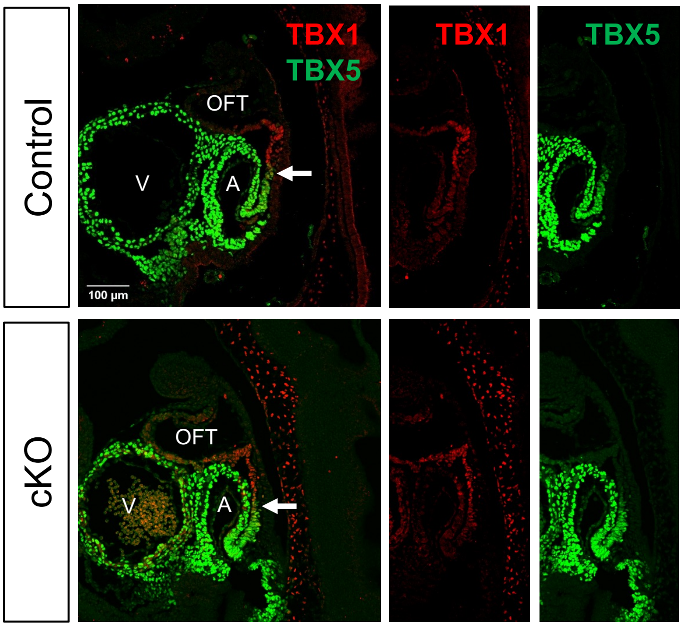

Supplemental Figure S6: Expression pattern of TBX1-TBX5 at the dorsal pericardial wall is unaltered by the loss of *Chd7*.

Immunofluorescence on medial sagittal sections of control and *Chd7* cKO embryos using anti-TBX1 (red) and anti-TBX5 (green) antibodies. The arrow indicates the posterior limit of TBX1 and the anterior limit of TBX5 in the DPW. n=6.

Scale bar 100µm. OFT, outflow tract; a, atrium; v, ventricle.

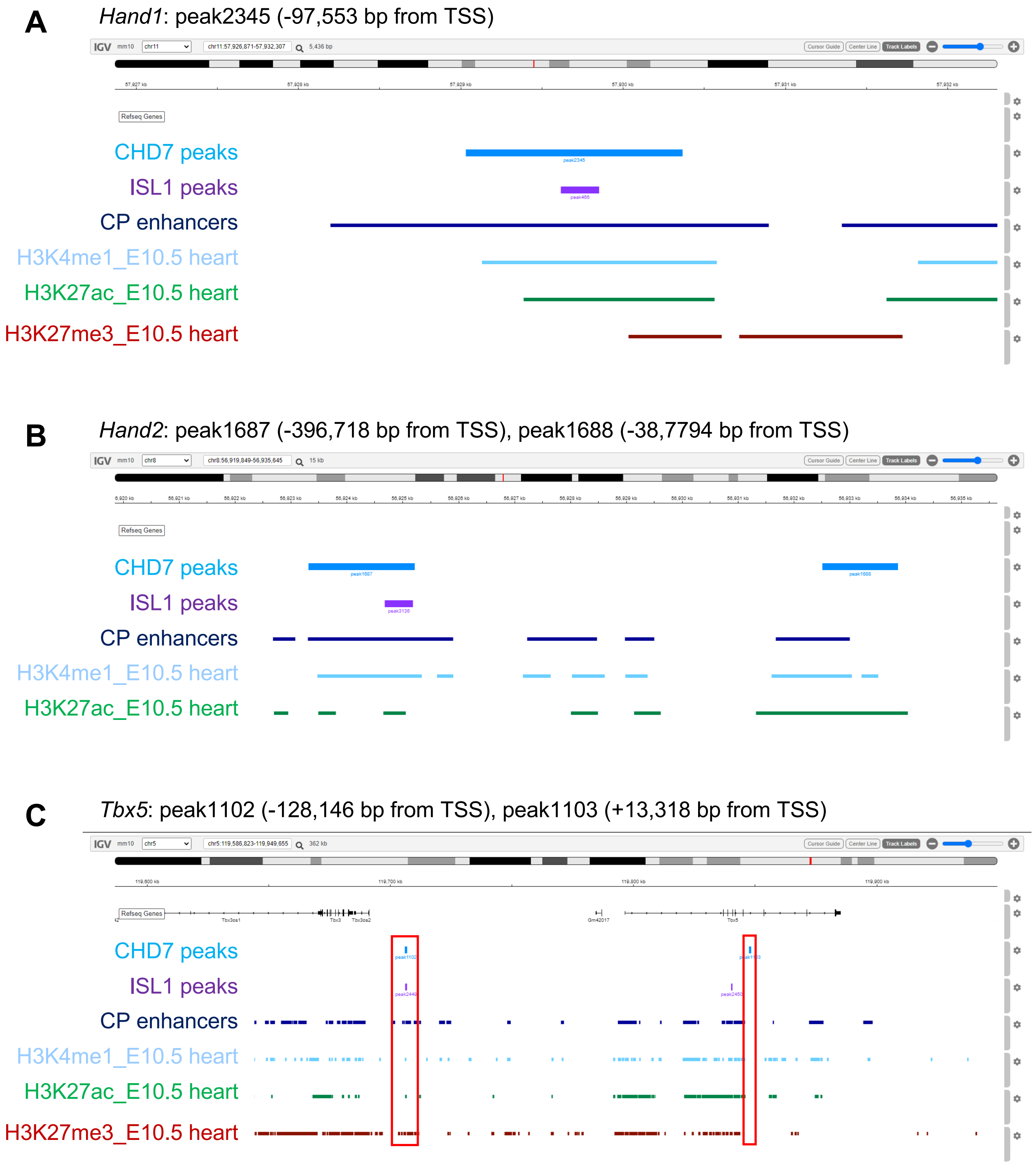

Supplemental Figure S7, related to figure 5: CHD7 binds cardiac enhancers and ISL1-bound regions near cardiac TFs deregulated in Chd7 cKO embryos.
Genome browser snapshots including CHD7 peaks (mid blue), ISL1 peaks (purple), CP enhancers (dark blue), H3K4me1 (light blue), H3K27ac (green) and H3K27me3 (dark red) from E10.5 hearts at *Hand1* (A) and *Hand2* (B) and *Tbx5* (C) loci.
Details on the tracks used can be found in the methods section.

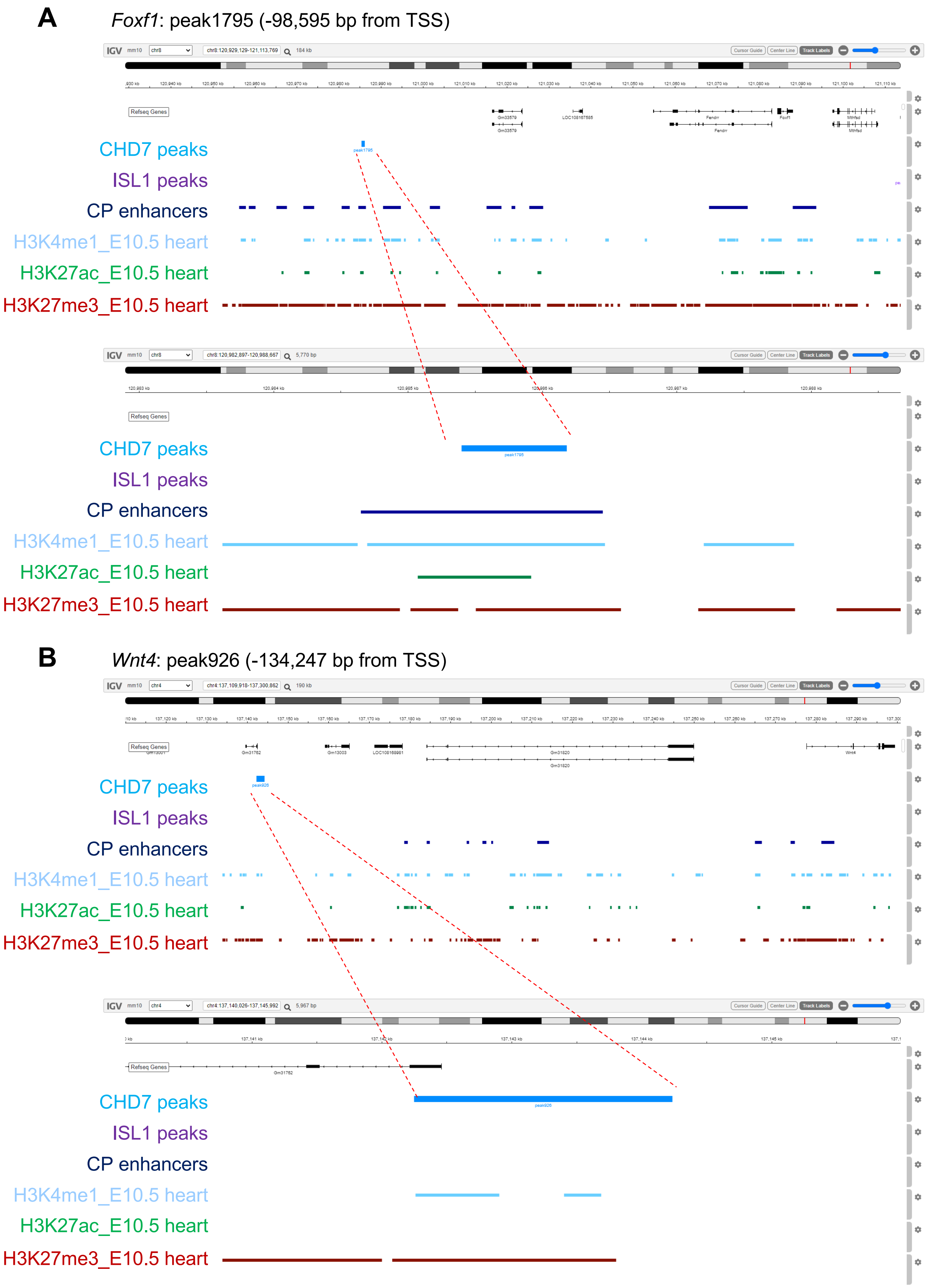

Supplemental Figure S8, related to figure 5: CHD7 binds cardiac TFs upregulated in *Chd7* cKO embryos. Genome browser snapshots at *Foxf1* (A) and *Wnt4* (B) loci showing tracks with CHD7 peaks (mid blue), ISL1 peaks (purple), CP enhancers (dark blue), H3K4me1 (light blue), H3K27ac (green) and H3K27me3 (dark red) from E10.5 hearts.
Details on the tracks used can be found in the methods section.

A **CHD7 CUT&RUN motif (RSAT): all peaks**

Motif	Logo	3 Top hits in databases
oligos_6nt_mkv4_m1oligos_6nt_mkv4_m1	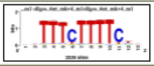	versus Homer: homer_280_T1ISRE, homer_234_CGGAAGTGAAAC, homer_109_Gata6, e-value=1.8e-21
oligos_6nt_mkv4_m2oligos_6nt_mkv4_m2	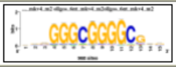	versus Homer: homer_266_Sp1, homer_146_KLF3, homer_145_KLF14, e-value=8.9e-21
oligos_6nt_mkv4_m3oligos_6nt_mkv4_m3	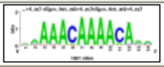	versus Homer: homer_5_AGTAAACAAAAAAGAACANA, e-value=4e-18
oligos_6nt_mkv4_m4oligos_6nt_mkv4_m4	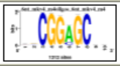	versus Homer: homer_266_Sp1, homer_326_ZNF519, e-value=6.3e-13
oligos_6nt_mkv4_m5oligos_6nt_mkv4_m5	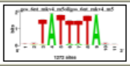	versus Homer: homer_163_Mef2c, homer_161_Mef2a, homer_30_Cdx2, e-value=9.8e-12
positions_6ntpositions_6nt_m1		versus Homer: no match
oligos_7nt_mkv5_m1oligos_7nt_mkv5_m1	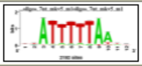	versus Homer: homer_125_Hnf1, homer_163_Mef2c, homer_164_Mef2d, e-value=9.1e-20
oligos_7nt_mkv5_m2oligos_7nt_mkv5_m2	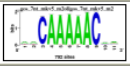	versus Homer: no match e-value=6e-14
oligos_7nt_mkv5_m3oligos_7nt_mkv5_m3	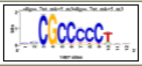	versus Homer: homer_63_Egr2, homer_145_KLF14, homer_62_Egr1, e-value=1.6e-12
oligos_7nt_mkv5_m4oligos_7nt_mkv5_m4	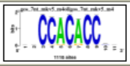	versus Homer: homer_65_EKLF, homer_147_Klf4, homer_148_KLF5, e-value=6.9e-12
oligos_7nt_mkv5_m5oligos_7nt_mkv5_m5	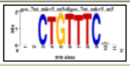	versus Homer: homer_78_AGGAAACAGCTG, homer_97_FOXP1, homer_316_ZNF189, e-value=7.9e-10

B

Enrichr: RNAseq Automatic GEO Signatures Mouse Down		
Term: Pioneering Isl1 Cardiomyocyte Fate GSE80383 1		
	'SHF'	'HEART'
**P-value**	8.80E-39	1.47E-24
**Adjusted P-value**	1.74E-35	8.79E-22
**Odds Ratio**	22.71214207	9.867209346
**Genes**	MYOM1;PARM1;HSPB7;SIX1;SLC8A1;TTN;RGS4;RGS5;CSRP3;3632451O06RIK;LBH;MECOM;CAPN6;ERBB4;XIRP1;TNNI1;SMYD1;SOX6;SH3BGR;MYBPC3;MYBPC1;MEF2C;JAG1;PRRX1;ACTN2;TNNC1;SEMA6D;SORBS2;ISL1;MYL4;VCAN;MYL7;ACTC1;MYL2;TNNT2;CDHR1;HAND2;MYL3;CDO1;ASB2;DGKK;MYH6;MYH7	FAM49A;SLC24A2;SMPX;LAMA2;SYNPO2;HSPB7;TWIST1;ABAT;CACNA1D;THBS4;RGS4;CSRP3;3632451O06RIK;SCUBE1;IGFBPL1;PLN;ERBB4;CMYA5;PCDHAC2;PRKG1;MEF2C;EPHA7;MYBPC1;PRRX1;ANGPT1;ACTN2;ACSL1;NEBL;SEMA6D;KLHL24;TECRL;OBSCN;MYL1;KITL;HAND2;PPP1R3D;MAB21L2;HRC;RARB;CDO1;HCN1

C **'SHF' DEGs with CHD7 binding**

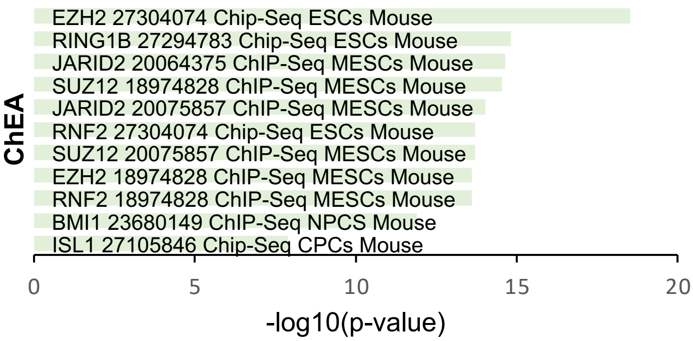

D **'HEART' DEGs with CHD7 binding**

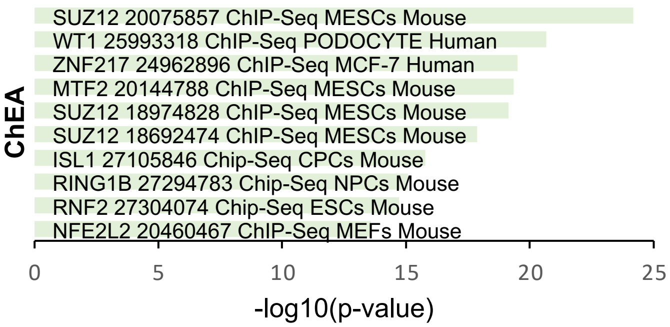

Supplemental Figure S9, related to figure 6: Further characterisation of CHD7 binding sites/direct targets.
(A) Motifs enrichment analysis of CHD7 peaks using RSAT peak-motifs. Discovered motifs were compared with known motifs from Homer. e-value is the adjusted p-value (corrected for multiple testing, calculated by RSAT peak-motifs software).
(B) Significant overlap between DEGs down-regulated in 'SHF' and 'HEART' and genes down-regulated in *Isl1*$^{-/-}$ CPCs (dataset GSE80383), based on the 'RNAseq Automatic GEO Signatures Mouse Down' function of Enrichr. P-value, adjusted p-value, odds ratio (all calculated by Enrichr) and common genes are displayed for each comparison.
Transcription factors bound at the promoters of 'SHF' (C) or 'HEART' (D) DEGs with CHD7 binding, based on the ChEA function of Enrichr (p-value from Fisher exact test).

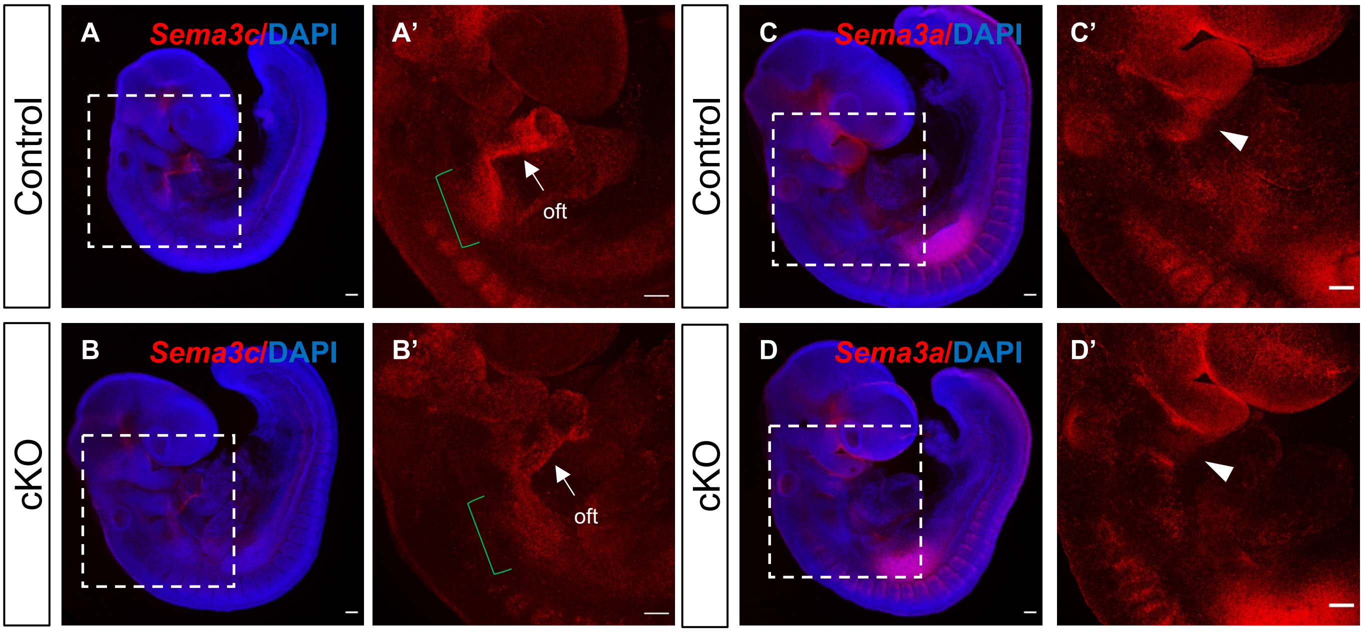

E. Relative Quantification of fluorescence intensity

Gene	ROI quantified	Control mean	control STDEV	Chd7 cKO mean	cKO STDEV	Fold Change (cKO/control)	p-value (TTEST)	Result
Sema3c	OFT	147284	11868	111132	10837	**0.75**	0.0080	Reduced
Sema3c	SHF region	188179	23736	152037	4536	**0.81**	0.0412	Reduced
Sema3a	BA2	168507	21494	118656	25739	**0.70**	0.0421	Reduced
Sema3a	SHF	127175	14452	113347	14528	**0.89**	0.2868	Non significant

Supplemental Figure S10: Expression of *Sema3c* and *Sema3a* is reduced in *Chd7* cKO embryos.
Whole mount in situ HCR staining of control and *Chd7* cKO embryos at E9.5 for *Sema3c* (A-B''') and *Sema3a* (C-D'''). The embryos are presented as maximum z projection. n = 4.
Scale bars, 100µm. Green bracket shows the SHF region; oft, outflow tract; arrowheads indicate cells in branchial arch 2 (BA2).
(E) Relative quantification of fluorescence intensity in selected regions of interest (ROI).

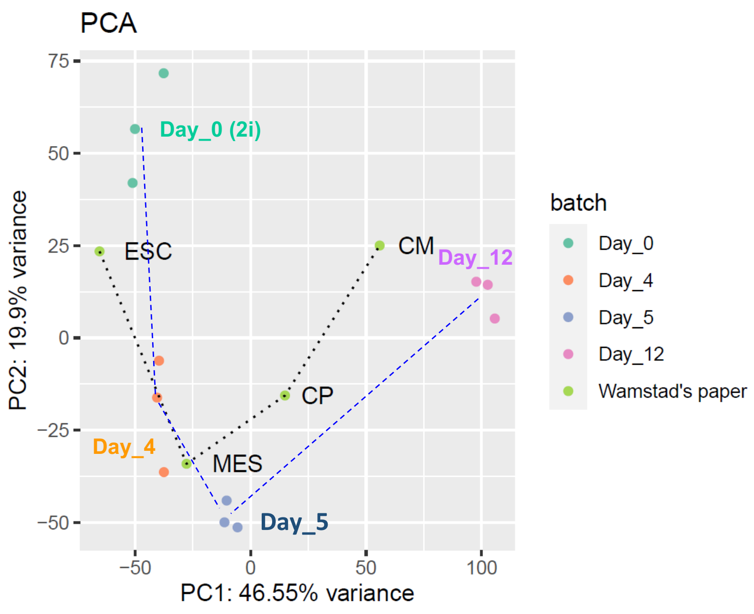

Supplemental figure S11: Our *in vitro* CM differentiation follows a similar trajectory as Wamstad et al[37] CM differentiation.
Principal component analysis (PCA) of our *in vitro* differentiation timepoints compared with *in vitro* CM differentiation time points from Wamstad et al[37] based on RNA-seq data. Please note our starting ES cells (Day_0) were cultured in 2i serum-free conditions. For Wamstad et al timepoints ESC: embryonic stem cell (day 0), MES: mesoderm (day 4), CP: cardiac precursor (day 5.3; 8 hours after our day 5), and CM: cardiomyocyte (day 10). Dashed lines indicate the differentiation trajectory in our (blue) and Wamstad et al (black) CM differentiations.

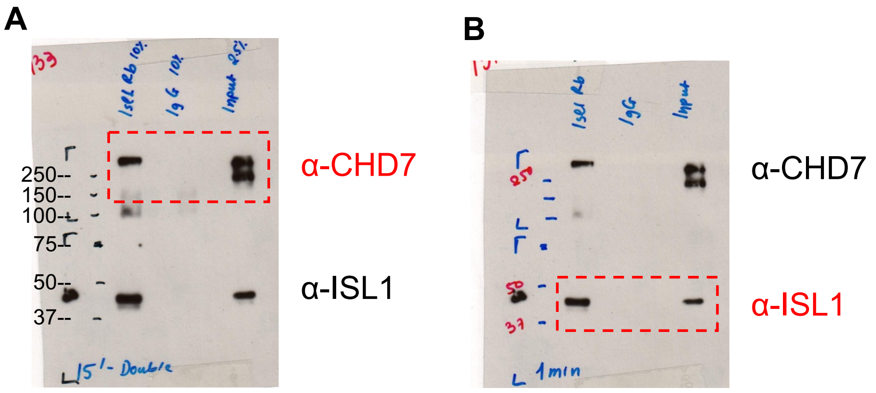

Supplemental Figure S12. Uncropped western blot images.
Gels corresponding to figure 6G (α-CHD7 from A and α-ISL1 from B).
