# Supplementary material for: CHARGE syndrome-associated CHD7 acts at ISL1-regulated enhancers to modulate second heart field gene expression
Source: Cardiovasc Res. 2023 Apr 13;119(11):2089–105. doi: 10.1093/cvr/cvad059 (PMC10478754; doi:10.1093/cvr/cvad059)
Supplement: cvad059_Supplementary_Data [file cvad059_supplementary_data.zip › Supplementary Material_all_updated.docx]

**Title:**

CHARGE syndrome-associated CHD7 acts at ISL1-regulated enhancers to modulate second heart field gene expression

**Short title:**

CHD7 regulates a cardiogenic GRN in mesoderm

Athanasia Stathopoulou^1, #^, Ping Wang^2, 3^, Charlotte Thellier^4^, Robert G Kelly^4^, Deyou Zheng^2, 5^, Peter J Scambler^1^

1. Developmental Biology of Birth Defects, UCL Great Ormond Street Institute of Child Health, 30 Guilford Street, London WC1N 1EH, UK.

2. Department of Genetics, Albert Einstein College of Medicine, Bronx, NY, USA.

3. School of Medical Imaging, Tianjin Medical University, Tianjin, China.

4. Aix-Marseille University, CNRS UMR 7288, IBDM, Marseille, France.

5. Departments of Neurology and Neurosciences, Albert Einstein College of Medicine, Bronx, NY, USA.

# Corresponding email: a.stathopoulou@ucl.ac.uk

**Manuscript category:** Original Article

| **Table S1: Relative Quantification of fluorescence intensity** | | | | | | | | | |
| --- | --- | --- | --- | --- | --- | --- | --- | --- | --- |
| **Gene** | **ROI quantified** | **Control mean** | control STDEV | **Chd7 cKO mean** | cKO STDEV | **Fold Change (cKO/control)** | **p-value (TTEST)** | **Relevant Figures** | **Result** |
| ***Mef2c*** | DPW | 217851 | 24566 | 141477 | 11638 | **0.65** | 0.0165 | Fig. 3 and S3 | Reduced |
| ***Mef2c*** | BA2 | 225707 | 8054 | 192778 | 13717 | **0.85** | 0.0429 |  | Reduced |
| ***Fgf10*** | DPW | 299688 | 40391 | 229163 | 22803 | **0.76** | 0.0438 |  | Reduced |
| ***Isl1*** | BA2 | 67713 | 5775 | 56104 | 5349 | **0.83** | 0.0432 |  | Reduced |
| ***Isl1*** | SHF+ proximal OFT | 313317 | 36287 | 294604 | 36449 | **0.94** | 0.5518 |  | Non significant |
| ***Hand2*** | SHF+ proximal OFT | 348528 | 32177 | 251199 | 27652 | **0.72** | 0.0073 | Fig. S4 | Reduced |
| ***Hand2*** | OFT+BA2 | 412552 | 48722 | 308628 | 50720 | **0.75** | 0.0429 |  | Reduced |
| ***Hand1*** | OFT+BA2 | 249606 | 7534 | 220232 | 18735 | **0.88** | 0.0453 |  | Reduced |
| ***Foxf1*** | pSHF | 169225 | 8327 | 205755 | 13247 | **1.22** | 0.0130 | Fig. 4 | Increased |
| ***Tbx5*** | pSHF | 202850 | 22726 | 323442 | 57961 | **1.59** | 0.0153 |  | Increased |
| ***Osr1*** | pSHF | 390403 | 36337 | 652616 | 76971 | **1.67** | 0.0018 |  | Increased |
| ***Wnt4*** | pSHF | 264103 | 56999 | 376463 | 46334 | **1.43** | 0.0381 |  | Increased |
| ***Tbx1*** | BA2 | 188807 | 22127 | 179588 | 22574 | **0.95** | 0.6314 | Fig. S5 | Non significant |
| ***Tbx1*** | SHF (including MLPs) | 221533 | 17927 | 199790 | 9427 | **0.90** | 0.1123 |  | Non significant |
| ***Aplnr*** | MLPs | 285086 | 38877 | 264383 | 45549 | **0.93** | 0.5712 |  | Non significant |
| **Internal control tissues** | | | | | | | | | |
| ***Isl1*** | motor neurons @ somite 2 | 148597 | 16524 | 170160 | 20197 | **1.15** | 0.2023 | Fig. 3 | Non significant |
| ***Isl1*** | motor neurons @ somite 3 | 144268 | 13462 | 169528 | 14115 | **1.18** | 0.0661 |  | Non significant |
| ***Fgf10*** | FL | 341528 | 58407 | 424235 | 59372 | **1.24** | 0.1362 |  | Non significant |
| ***Fgf10*** | OV | 342824 | 48035 | 350297 | 34415 | **1.02** | 0.8339 |  | Non significant |
| ***Tbx5*** | FL | 354330 | 66059 | 366933 | 82018 | **1.04** | 0.8426 | Fig. 4 | Non significant |
| ***Mef2c*** | 1st somite | 155024 | 15984 | 136814 | 14617 | **0.88** | 0.3002 | Fig. 3 | Non significant |
| ***Mef2c*** | 2nd somite | 166276 | 40399 | 150474 | 26808 | **0.90** | 0.6688 |  | Non significant |

| **Table S2: Selected DEGs in *Chd7* cKO embryos** | | | |
| --- | --- | --- | --- |
| **Gene** | ***Chd7* cKO**  **‘SHF’** | ***Chd7* cKO**  **‘HEART’** | **Chd7 CUT&RUN peaks (distance to TSS, bp)** |
| ***Fgf10*** | down | down | peak2899 (-119853), peak2900 (+44446) |
| ***Isl1*** | down | ns | peak2895 (-199836) |
| ***Hand1*** | down | down | peak2345 (-97553) |
| ***Hand2*** | down | down | peak1687 (-396718), peak1688 (-387794) |
| ***Mef2c*** | down | down | NA |
| ***Tbx5*** | up | NS | peak1102 (-128146), peak1103 (+13318) |
| ***Wnt4*** | up | NS | peak926 (-134247) |
| ***Osr1*** | up | NS | peak2557 (-320864), peak2558 (-180227) |
| ***Foxf1*** | up | NS | peak1795 (-98595) |
| ***Tbx1*** | NS | NS | NA |
| ***Aplnr*** | NS | NS | NA |

**SUPPLEMENTAL FIGURE LEGENDS**

**Supplemental figure S1, related to figure 1: Reduced CM markers in unbiased analysis of *Chd7* cKO ‘SHF’ downregulated genes.**

(A) Bar graph showing the numbers of DEGs in ‘SHF’ and ‘HEART’ tissue of *Chd7* cKO embryos.

(B, D) GO terms enriched in genes up-regulated in *Chd7* cKO ‘SHF’ (B) and ‘HEART’ (D) ranked by p-value from the Fisher exact test, by Enrichr.

(C) Comparison of down-regulated genes in *Chd7* cKO ‘SHF’ with previously published single cell RNA-seq data from Mesp1-expressing cells^32^. Clusters of the different cell types are shown at the bottom of the heatmap and they are the same as in the original publication. Selected genes enriched in CM clusters are presented on the right and left side of the heatmap. Row indicates the mean gene expression in each cluster (blue to white, to red; low to high).

(E) Venn diagram showing the overlap of genes up- or down-regulated in cKO ‘SHF’ and ‘HEART’.

(F) GO:cellular component analysis of common DEGs in cKO ‘HEART’ and E11.5 hearts, related to figure 1I.

**Supplemental figure S2, related to figure 2: CHD7 direct targets.**

(A) Timeline of cardiomyocyte differentiation protocol, showing ‘key’ stages described by Wamstad et al ^37^. ESC, embryonic stem cell; MES, mesoderm; CP, cardiac precursor and CM, cardiomyocyte. Based on PCA (figure 2A) we selected “Day 5” as the appropriate stage for the in vivo ‘SHF’ samples. “Day 5” is exactly 24 hours after the MES stage at day 4, when the cells are plated onto gelatin-coated plates.

(B) Bar graph showing the number of genes associated with CHD7 peaks.

(C) Bioplanet pathways enriched in genes associated with CHD7 peaks. Pathways enriched in ‘SHF’ (D) or ‘HEART’ (F) DEGs with CHD7 binding based on WikiPathway human enrichment. Terms are ranked by p-values from the Fisher exact test, by Enrichr.

(E) Venn diagram displaying overlap of CHD7 peaks with downregulated (down) and upregulated (up) genes in ‘SHF’. p-values are from hypergeometric test.

(G) GO biological processed enriched in genes downregulated in ‘HEART’ with CHD7 binding, ranked by p-values (from Fisher exact test).

**Supplemental Figure S3, related to figures 3 and 4: Additional views and ‘virtual’ sections of the embryos presented in figures 3 and 4.**

Whole mount *in situ* HCR staining of control and *Chd7* cKO (*Mesp1-Cre; Chd7^fl/fl^*) embryos at E9-9.5 for *Fgf10* (A-B’’’), *Mef2c* (C-D’’’), *Isl1* (E-F’) and Tbx5 (G-H’). Some of the thick ‘virtual’ sections are also presented in the main figures. Boxed regions in are shown in neighbouring panels.

Confocal maximum projection of selected lateral or medial z-stacks are displayed, with the number of stacks used indicated on top of each image. The ‘step’ between the different z-stacks is 3.25μm. n=4.

Arrowheads indicate cells in branchial arches, green bracket shows the SHF region. SHF, second heart field; DPW, dorsal pericardial wall. Scale bars represent 100μm.

**Supplemental Figure S4: Expression of *Hand1* and *Hand2* is reduced in *Chd7* cKO embryos.**

Whole mount in situ HCR staining of control and *Chd7* cKO embryos at E9.5 for *Hand2* (A-B’’’) and *Hand1* (C-D’’’). The entire embryo and selected lateral and medial stacks are presented as maximum z projection. n = 4.

Scale bars, 100μm. DPW, dorsal pericardial wall; oft, outflow tract.

**Supplemental Figure S5: Distribution and expression of *Tbx1* and *Aplnr* is not altered in *Chd7* cKO embryos.**

Whole mount *in situ* HCR staining for *Tbx1* (A-B’) and *Aplnr* (C-D’) in control and *Chd7* cKO embryos. Maximum intensity projections of the entire embryos are presented. Yellow arrows highlight the MLP cells. n=4.

Scale bars,100μm. ov, otic vesicle; MLPs, multilineage primed progenitors.

**Supplemental Figure S6: Expression pattern of TBX1-TBX5 at the dorsal pericardial wall is unaltered by the loss of *Chd7*.**

Immunofluorescence on medial sagittal sections of control and *Chd7* cKO embryos using anti-TBX1 (red) and anti-TBX5 (green) antibodies. The arrow indicates the posterior limit of TBX1 and the anterior limit of TBX5 in the DPW. n=6.

Scale bar 100μm. OFT, outflow tract; a, atrium; v, ventricle.

**Supplemental Figure S7, related to figure 5: CHD7 binds cardiac enhancers and ISL1-bound regions near cardiac TFs deregulated in Chd7 cKO embryos.**

Genome browser snapshots including CHD7 peaks (mid blue), ISL1 peaks (purple), CP enhancers (dark blue), H3K4me1 (light blue), H3K27ac (green) and H3K27me3 (dark red) from E10.5 hearts at *Hand1* (A) and *Hand2* (B) and *Tbx5* (C) loci.

Details on the tracks used can be found in the methods section.

**Supplemental Figure S8, related to figure 5: CHD7 binds cardiac TFs upregulated in *Chd7* cKO embryos.**

Genome browser snapshots at *Foxf1* (A) and *Wnt4* (B) loci showing tracks with CHD7 peaks (mid blue), ISL1 peaks (purple), CP enhancers (dark blue), H3K4me1 (light blue), H3K27ac (green) and H3K27me3 (dark red) from E10.5 hearts.

Details on the tracks used can be found in the methods section.

**Supplemental Figure S9, related to figure 6: Further characterisation of CHD7 binding sites/direct targets.**

(A) Motifs enrichment analysis of CHD7 peaks using RSAT peak-motifs. Discovered motifs were compared with known motifs from Homer. e-value is the adjusted p-value (corrected for multiple testing, calculated by RSAT peak-motifs software).

(B) Significant overlap between DEGs down-regulated in ‘SHF’ and ‘HEART’ and genes down-regulated in *Isl1^-/-^* CPCs (dataset GSE80383), based on the ‘RNAseq Automatic GEO Signatures Mouse Down’ function of Enrichr. P-value, adjusted p-value, odds ratio (all calculated by Enrichr) and common genes are displayed for each comparison.

Transcription factors bound at the promoters of ‘SHF’ (C) or ‘HEART’ (D) DEGs with CHD7 binding, based on the ChEA function of Enrichr (p-value from Fisher exact test).

**Supplemental Figure S10: Expression of *Sema3c* and *Sema3a* is reduced in *Chd7* cKO embryos.**

Whole mount in situ HCR staining of control and *Chd7* cKO embryos at E9.5 for *Sema3c* (A-B’’’) and *Sema3a* (C-D’’’). The embryos are presented as maximum z projection. n = 4.

Scale bars, 100μm. Green bracket shows the SHF region; oft, outflow tract; arrowheads indicate cells in branchial arch 2 (BA2).

(E) Relative quantification of fluorescence intensity in selected regions of interest (ROI).

**Supplemental figure S11: Our *in vitro* CM differentiation follows a similar trajectory as Wamstad et al^37^ CM differentiation.**

Principal component analysis (PCA) of our *in vitro* differentiation timepoints compared with *in vitro* CM differentiation time points from Wamstad et al^37^ based on RNA-seq data. Please note our starting ES cells (Day_0) were cultured in 2i serum-free conditions. For Wamstad et al timepoints ESC: embryonic stem cell (day 0), MES: mesoderm (day 4), CP: cardiac precursor (day 5.3; 8 hours after our day 5), and CM: cardiomyocyte (day 10). Dashed lines indicate the differentiation trajectory in our (blue) and Wamstad et al (black) CM differentiations.

**Supplemental Figure S12. Uncropped western blot images.**

Gels corresponding to figure 6G (α-CHD7 from A and α-ISL1 from B).

**SUPPLEMENTARY METHODS**

**Additional bioinformatics analysis**

**Comparison of RNA-seq samples to *in vitro* differentiating samples**

Normalized FPKMs from ‘SHF’, ‘HEART’ and samples in Wamstad’s paper ^1^ were used for PCA analysis, without additional batch correction. We used the function prcomp for PCA and ggplot for visualization in R.

**Comparison with single cell *Mesp1***

Gene expression in recently published scRNA-seq data for the E9.5 *Mesp1* lineage^2^ was analyzed for the significantly up- and down-regulated genes in *Chd7* cKO ‘SHF’ vs controls. Cell type annotation by the original authors were used to compute average gene expression for each gene in the wild type ‘SHF’. The data were then clustered to compare the expression of the up- or down-regulated genes across cell types and presented as a heatmap.

**CUT&RUN data analysis**

Deeptools^3^ (v3.1.0) was used to compute read coverage across the peaks and generate heatmap for comparing the coverage across samples.

Association of the CUT&RUN peaks with genes was performed using GREAT^4^, with default settings for mm10. HOMER (v4.7, default setting) was used for motif enrichment analysis for all the peaks or the peak subsets mapped to up- or down-regulated genes in the *Chd7* cKO. To determine what TFs may co-occupy with CHD7, the CUT&RUN peaks were compared to a database of published ChIP-seq data using BART^5^. The ISL1 ChIP-seq peaks from a previous study ^6^ were obtained and those overlapping with CHD7 CUT&RUN by at least 1 bp were determined after they were mapped to the mm10.

Distribution of distance from peaks to nearest TSS was calculated using ChIP-Enrich ^7^.

**Extended bioinformatics analysis**

Gene ontology (GO) and pathways analysis was performed using Enrichr ^8-10^.

Intersection of gene lists was performed using Venny (<https://bioinfogp.cnb.csic.es/tools/venny/>). The statistical significance of the overlap between two gene lists was calculated using the calculator in <http://nemates.org/MA/progs/overlap_stats.html>, adjusted for the number of genes in the mouse genome. All the gene list overlaps presented had representation factor >1, which indicates more overlap than expected of two independent groups. The hypergeometric probability p-value is provided in the relevant figures.

IGV genome browser ^11^ was used for illustration of CHD7 binding sites, with the addition of the following published datasets: CP enhancers ^1^, ISL1 binding sites ^6^, and H3K4me1, H3K27ac and H3K27me3 ChIP-seq datasets from E10.5 hearts (by Bing Ren, part of the ENCODE project ^12^). Datasets mapped onto mm9 were converted to mm10 using UCSC LiftOver.

**CUT&RUN sequencing, library preparation**

Library preparation was performed with the NEB DNA Ultra II assay with the following deviations;

- A-tailing temperature was reduced to 50ºC and performed for an hour.
- Full length UDI-UMI adapters (IDT) were diluted to 0.3µM for ligation.
- No USER linearisation step was performed.
- After ligation, 1.1X volume of SPRIselect was used for clean-up.
- During PCR, anneal/extension time was cut to 13 seconds to exclude amplification of large fragments.
- An additional 1X volume SPRI select clean-up was performed post PCR to remove adapter-dimer.

**HCR whole mount image acquisition and analysis**

Images were acquired using a Zeiss LSM 880 Upright Confocal Multiphoton microscope, with 10x/NA0.5 W-Plan Apochromat Water dipping objective or 5x/NA0.16 EC Plan-Neofluar objective. Images were saved as czi files and stacks were exported in FIJI. Using FIJI software MAX intensity projection method of selected Z stacks was used to produce the images presented. All the control and cKO embryos of each HCR probe were imaged using the same settings, on the same imaging session.

For quantification of relative fluorescence intensity SUM slices projection method was used to apply a Z projection to the stack of images. Equal number of Z stacks were projected from control and cKO embryos and Measure function in FIJI was used for the quantification. The ‘mean gray value’ of the region of interest (ROI) marked by a rectangle was measured in all embryos tested. The average of the ‘mean gray value’ of control and cKO embryos was calculated and the ratio of cKO to control was used to identify gene expression changes (fold change cKO/control). Two-tailed student’s t-test was performed to determine statistical significant differences between control and cKO embryos. Data is presented in table S1.

**Immunostaining**

Immunofluorescence on paraffin sections was performed as previously described ^13^, using the following antibodies: rabbit anti-TBX1 (1/100, Lifescience Ls-C31179), mouse anti-TBX5 (1/100, Santa Cruz, Sc-515536) and secondary antibodies raised in donkey (Life Science technologies). Images were acquired using a Zeiss confocal LSM780 microscope with a 20X objective.

**REFERENCES for Supplementary Methods**

1. Wamstad JA, Alexander JM, Truty RM, Shrikumar A, Li F, Eilertson KE, Ding H, Wylie JN, Pico AR, Capra JA, et al. Dynamic and coordinated epigenetic regulation of developmental transitions in the cardiac lineage. *Cell*. 2012;151:206-220. doi: 10.1016/j.cell.2012.07.035

2. Nomaru H, Liu Y, De Bono C, Righelli D, Cirino A, Wang W, Song H, Racedo SE, Dantas AG, Zhang L, et al. Single cell multi-omic analysis identifies a Tbx1-dependent multilineage primed population in murine cardiopharyngeal mesoderm. *Nat Commun*. 2021;12:6645. doi: 10.1038/s41467-021-26966-6

3. Ramirez F, Ryan DP, Gruning B, Bhardwaj V, Kilpert F, Richter AS, Heyne S, Dundar F, Manke T. deepTools2: a next generation web server for deep-sequencing data analysis. *Nucleic Acids Res*. 2016;44:W160-165. doi: 10.1093/nar/gkw257

4. McLean CY, Bristor D, Hiller M, Clarke SL, Schaar BT, Lowe CB, Wenger AM, Bejerano G. GREAT improves functional interpretation of cis-regulatory regions. *Nat Biotechnol*. 2010;28:495-501. doi: 10.1038/nbt.1630

5. Ma W, Wang Z, Zhang Y, Magee NE, Feng Y, Shi R, Chen Y, Zang C. BARTweb: a web server for transcriptional regulator association analysis. *NAR Genom Bioinform*. 2021;3:lqab022. doi: 10.1093/nargab/lqab022

6. Wang Y, Li Y, Guo C, Lu Q, Wang W, Jia Z, Chen P, Ma K, Reinberg D, Zhou C. ISL1 and JMJD3 synergistically control cardiac differentiation of embryonic stem cells. *Nucleic Acids Res*. 2016;44:6741-6755. doi: 10.1093/nar/gkw301

7. Welch RP, Lee C, Imbriano PM, Patil S, Weymouth TE, Smith RA, Scott LJ, Sartor MA. ChIP-Enrich: gene set enrichment testing for ChIP-seq data. *Nucleic Acids Res*. 2014;42:e105. doi: 10.1093/nar/gku463

8. Chen EY, Tan CM, Kou Y, Duan Q, Wang Z, Meirelles GV, Clark NR, Ma'ayan A. Enrichr: interactive and collaborative HTML5 gene list enrichment analysis tool. *BMC Bioinformatics*. 2013;14:128. doi: 10.1186/1471-2105-14-128

9. Kuleshov MV, Jones MR, Rouillard AD, Fernandez NF, Duan Q, Wang Z, Koplev S, Jenkins SL, Jagodnik KM, Lachmann A, et al. Enrichr: a comprehensive gene set enrichment analysis web server 2016 update. *Nucleic Acids Res*. 2016;44:W90-97. doi: 10.1093/nar/gkw377

10. Xie Z, Bailey A, Kuleshov MV, Clarke DJB, Evangelista JE, Jenkins SL, Lachmann A, Wojciechowicz ML, Kropiwnicki E, Jagodnik KM, et al. Gene Set Knowledge Discovery with Enrichr. *Curr Protoc*. 2021;1:e90. doi: 10.1002/cpz1.90

11. Robinson JT, Thorvaldsdottir H, Winckler W, Guttman M, Lander ES, Getz G, Mesirov JP. Integrative genomics viewer. *Nat Biotechnol*. 2011;29:24-26. doi: 10.1038/nbt.1754

12. Consortium EP. An integrated encyclopedia of DNA elements in the human genome. *Nature*. 2012;489:57-74. doi: 10.1038/nature11247

13. De Bono C, Thellier C, Bertrand N, Sturny R, Jullian E, Cortes C, Stefanovic S, Zaffran S, Theveniau-Ruissy M, Kelly RG. T-box genes and retinoic acid signaling regulate the segregation of arterial and venous pole progenitor cells in the murine second heart field. *Hum Mol Genet*. 2018;27:3747-3760. doi: 10.1093/hmg/ddy266
